# Cooperative Wnt-Nodal Signals Regulate the Patterning of Anterior Neuroectoderm

**DOI:** 10.1371/journal.pgen.1006001

**Published:** 2016-04-21

**Authors:** Junko Yaguchi, Noriyo Takeda, Kazuo Inaba, Shunsuke Yaguchi

**Affiliations:** Shimoda Marine Research Center, University of Tsukuba, Shizuoka, Japan; Hopkins Marine Station, UNITED STATES

## Abstract

When early canonical Wnt is experimentally inhibited, sea urchin embryos embody the concept of a Default Model *in vivo* because most of the ectodermal cell fates are specified as anterior neuroectoderm. Using this model, we describe here how the combination of orthogonally functioning anteroposterior Wnt and dorsoventral Nodal signals and their targeting transcription factors, FoxQ2 and Homeobrain, regulates the precise patterning of normal neuroectoderm, of which serotonergic neurons are differentiated only at the dorsal/lateral edge. Loss-of-function experiments revealed that ventral Nodal is required for suppressing the serotonergic neural fate in the ventral side of the neuroectoderm through the maintenance of *foxQ2* and the repression of *homeobrain* expression. In addition, non-canonical Wnt suppressed *homeobrain* in the anterior end of the neuroectoderm, where serotonergic neurons are not differentiated. Canonical Wnt, however, suppresses *foxQ2* to promote neural differentiation. Therefore, the three-dimensionally complex patterning of the neuroectoderm is created by cooperative signals, which are essential for the formation of primary and secondary body axes during embryogenesis.

## Author Summary

The sea urchin embryo is similar to vertebrate embryos in that the default cell fate is potentially neurogenic, and normal development restricts the neural fate to the narrow area that locates at the anterior/dorsal region of the embryo. Because maintaining the default neural fate to the anterior/dorsal region is required for embryos to precisely integrate information from both the primary anterior-posterior and secondary dorsal-ventral body axes, these axes must be mutually linked by some mechanisms. In this study, we describe how the combination of orthogonally functioning signaling pathways regulates their targeting transcription factors expressing at the anterior neuroectoderm to restrict and pattern the default neurogenic region. By loss-of-function experiments using sea urchin embryos, we revealed that canonical and non-canonical Wnt pathways regulate the anterior neuroectoderm patterning along the primary axis, and TGF-ß signals control the patterning of the neuroectoderm along the secondary axis. In addition, we showed that the crosstalk between the Wnt and TGF-ß pathways was of importance in regulating the neuroectoderm patterning. As the default cell fate in some deuterostome embryos, including embryonic stem cells, is neurogenic, our findings could be widespread mechanisms to coordinate the remaining and/or suppressing developmental programs along different embryonic axes because Nodal and Wnt signals are critical in establishing early developmental polarities in many embryos.

## Introduction

Embryonic cells of some animals tend to be differentiated into neuroectoderm cells/neural progenitors unless they receive an extrinsic signal, so-called default model [1,2]. This characteristic is also applicable to mammalian embryonic stem cells and induced pluripotent cells (e.g., [3,4]). Therefore, normal development in such organisms can be rephrased as molecular mechanisms that repress the initial neuroectodermal fate and drive them to be differentiated into different cell types. Transforming growth factor-ß (TGF-ß) family members are one group of well-described signaling molecules that play essential roles in determining non-neuroectodermal cell fates. Among these, Chordin and Noggin, which were initially reported as neural inducers, function in protecting the initial neuroectodermal fate at the dorsal side in vertebrates from invading bone morphogenetic protein (BMP) signals that are expressed at the ventral side and that specify a non-neuroectodermal fate [2]. Wnts, another type of secreted signaling molecule, also have functions in repressing the initial anterior neuroectodermal fate. In vertebrates, posteriorly functioning Wnt inhibits anterior neuroectoderm specification genes, such as *otx2*, and leads to the specification of posterior neuroectoderm [5]. Together, these secreted signaling molecules that regulate body axis formation act to suppress the initial neuroectodermal fate during early embryogenesis. However, despite a large number of these non-neuroectodermal signals, embryos still maintain the neurogenic region in its proper size and location. In addition, within the remaining initial neurogenic ectoderm, each terminal cell differentiation is precisely controlled to organize the complicated neural network, i.e., the patterning of the neurogenic ectoderm is highly sophisticated in the restricted neuroectoderm of normal embryos.

**Fig 1:**
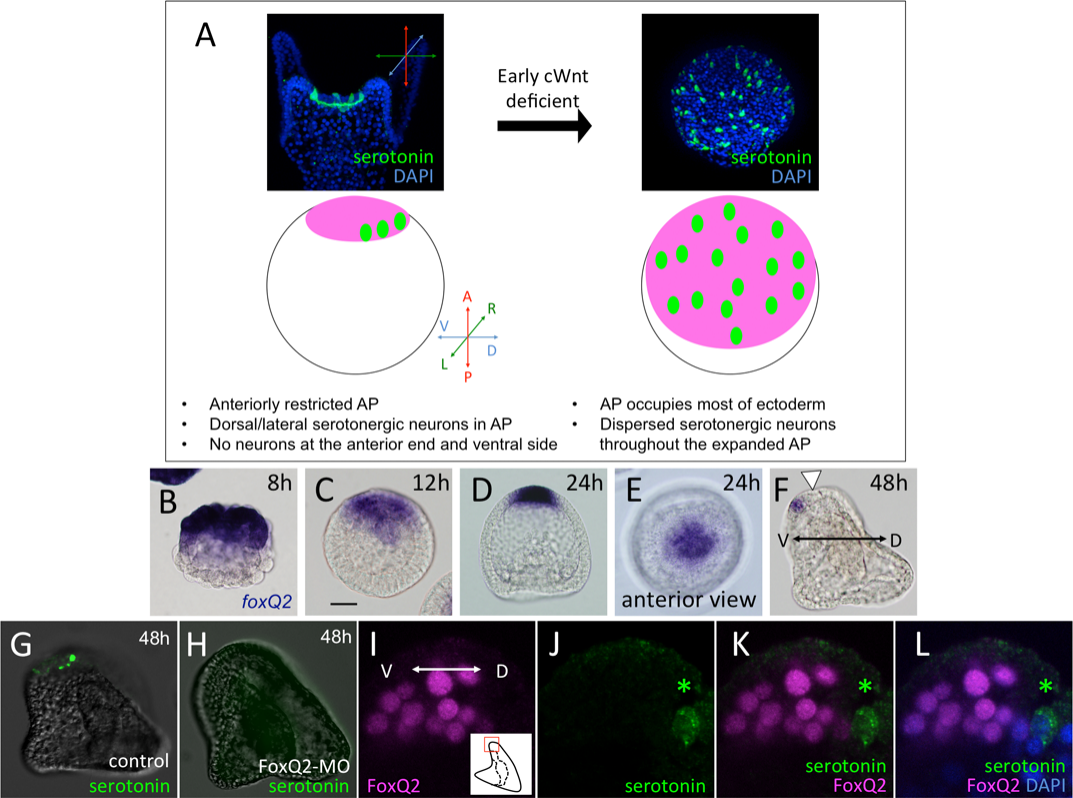
The pre-signaling state of most of the ectoderm is neurogenic and the region is patterned along the dorsal-ventral axis after restricted anteriorly. (A) A brief summary of the early cWnt-deficient embryonic phenotype, in which the initial (default) neuroectoderm covers most of the embryo and a number of serotonergic neurons are differentiated. In the drawings, the pink field and green spots indicate neuroectoderm and serotonergic neurons, respectively. (B-F) *foxQ2* patterns during the embryogenesis of the sea urchin, *Hemicentrotus pulcherrimus*. *foxQ2* is initially expressed at the anterior half (B) and gradually restricted to the anterior end by the blastula/gastrula stages (C-E). (F) *foxQ2* is expressed ventrally in the restricted AP region in the early pluteus stage. Left is ventral (V) and right is the dorsal (D) side. Arrowhead indicates the position where *foxQ2* gene expression is missing in the AP region. (G, H) FoxQ2 is required for the development of serotonergic neurons. Without FoxQ2, serotonergic neurons are not differentiated at 48 h (H) compared to control (G). (I-L) Sagittal section of the neuroectoderm field in a prism larva. Serotonergic neuron is differentiated at the dorsal edge of the AP and never includes FoxQ2 protein in its nucleus. Bar in (F) is 20 μm.

It has been suggested that the pre-signaling cell fate in sea urchin embryos is also anterior neuroectoderm, called the animal plate (AP). This is shown by an experiment in which the earliest canonical Wnt (cWnt) signal is inhibited by injecting the intracellular domain of cadherin (Δcad) to interfere with the nuclear localization of ß-catenin, resulting in most of the ectoderm of the injected embryos becoming specified as AP and differentiating into serotonergic neurons as well as other types of neurons and non-neural cells (Fig 1A: [6,7]). The expanded AP in the early cWnt-deficient embryos lacks patterning, and the serotonergic neurons are therefore dispersed throughout the AP. In contrast, the restricted AP in normal embryos differentiates into serotonergic neurons only at the dorsal/lateral edge, i.e., there are no serotonergic neurons observed at the ventral edge and central part (anterior end) of the AP (Fig 1A), even though most of the cells in the anteriorly restricted neurogenic region have the potential to be serotonergic neurons [8]. Nodal-BMP2/4, via Smad2/3-1/5/8 signaling along the dorsoventral axis, is one of the signaling networks that regulates the specification of cell fate and patterning in this region (reviewed in [9]), but their target transcription factors remain unclear. In summary, the developmental features of the AP of the sea urchin embryo are the following: 1) the serotonergic neural fate is executed only at the dorsal/lateral edge of the neuroectoderm, 2) the anterior end (i.e., the central part) of the AP does not differentiate serotonergic neurons, and 3) no serotonergic neurons appear at the ventral edge of the AP. Although information regarding the morphological and phenomenological characteristics of the development of the AP in sea urchin embryos has accumulated, the details of the molecular mechanisms that perform the intrinsic system of serotonergic neural fate specification at the dorsal/lateral edge of the neuroectoderm and that suppress the neural fate in other regions must still be defined. Thus, we have focused on the functional regulation between the signaling molecules and the transcription factors that control the patterning of the AP in the sea urchin embryo.

Serotonergic neurons in the sea urchin embryo are differentiated within the AP by nearly bilateral patterning (Fig 1A: [9,10]). Among the transcription factors that are zygotically expressed in the AP, the earliest is *foxQ2*. Based on its expression pattern and previous experimental data, FoxQ2 is present in all AP cells during early embryogenesis (Fig 1B–1E: [11,12]), and it is essential for the specification of most of the cell types in the AP region, including the serotonergic neurons and apical tuft of *Hemicentrotus pulcherrimus* (Fig 1G and 1H: [8,13]) and *Strongylocentrotus purpuratus* [12]. However, FoxQ2 mRNA disappears from the dorsal/lateral edge of the neuroectoderm, where the serotonergic neurons are differentiated (Fig 1F, arrowhead: [13]), and the protein cannot be detected in differentiating serotonergic neurons (Fig 1G–1J). In addition, FoxQ2 plays an essential role in the formation of the apical tuft cilia through the maintenance of the *ankAT-1* gene in later stages [14]. Because apical tuft cells are not serotonergic, these data suggest that FoxQ2 is first required for the specification of the most of the AP cells [12] but that the expression is subsequently suppressed in the cells at the dorsal/lateral edge of the AP, in which the serotonergic neural fate is executed. Thus, identifying the regulatory mechanisms of FoxQ2/*foxQ2* patterning along the dorsoventral axis must be one of the keys to understanding how the initial neurogenic ectoderm is patterned during sea urchin embryogenesis.

Homeobrain (Hbn: LC064116 for *Hemicentrotus pulcherrimus* Hbn) is a paired-like homeobox gene that is classified into the homeobrain-like (*hbnl*) family [15]. The gene expression patterns of *hbnl* family members have been reported in the fruit fly [16], sandworm [15], sea urchin [17,18] and sea anemone [19], but the *hbn* gene has not been identified in chordate genomes. The *hbn* expression pattern was first investigated in *Drosophila melanogaster*, where it initially appears in the anterior dorsal head primordium, which forms portions of the brain, and then in the ventral nerve cord during later stages. In sandworms (*Capitella* sp. I), *hbn* expression was detected in the developing brain, as in fruit flies, and in its larval eyes. In the sea anemone *Nematostella vectensis*, *hbn* is expressed throughout the blastoderm except for around the blastopore, and its expression is excluded from the aboral pole, where the apical tuft and the subsequent neurogenic region are formed. The expression pattern of *hbn* in sea urchin embryos was reported during the genome sequencing of *Strongylocentrotus purpuratus* [17,18,20,21]. In those studies, *hbn* was initially expressed in the animal pole region during the early blastula stage, and, at later stages, it appeared outside of and then disappeared from the AP, where *foxQ2* was expressed. Despite reports on the existence of the gene in some species, the control of its expression and its molecular function has not been investigated in all animals. Here, we focus on the function of Hbn because it is expressed in the same region as the *foxQ2* gene during the early specification stage of the AP. Then, we describe the roles of Hbn in the specification of serotonergic neurons and report that the regulation of *hbn* and *foxQ2* expression by Wnt and TGF-ß signals are essential for the precise patterning of the embryonic AP in the sea urchin *H*. *pulcherrimus*.

## Results

### *hbn* is required for the development of serotonergic neurons

**Fig 2:**
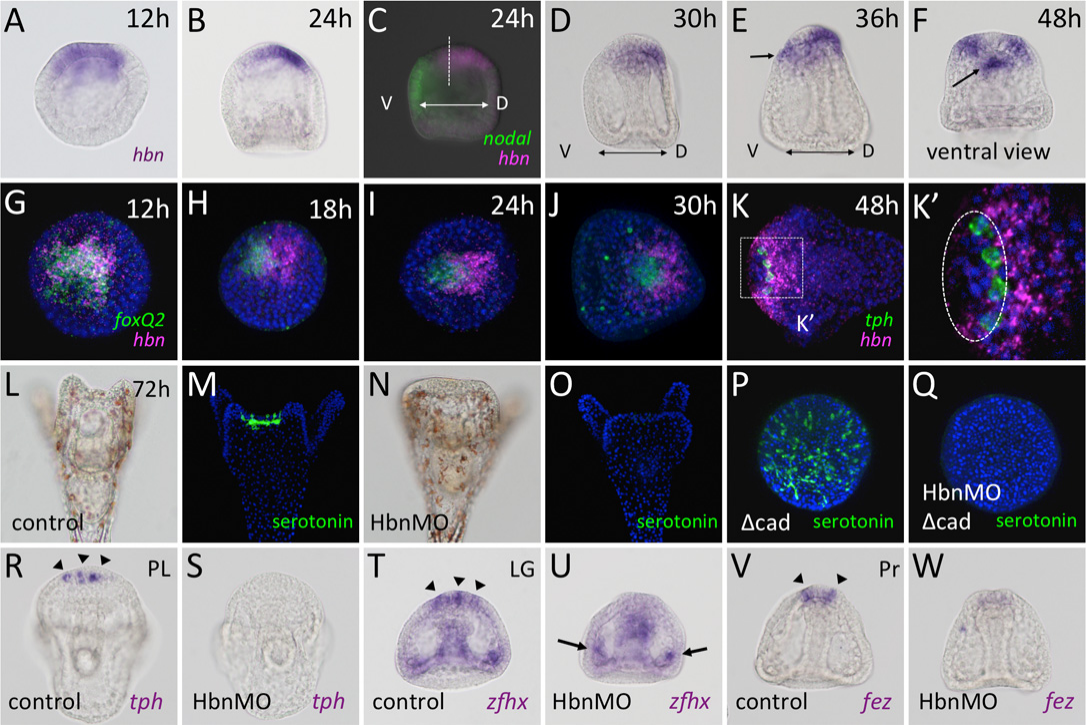
Homeobrain is required for the development of serotonergic neurons. (A-K’) Spatiotemporal expression pattern of *hbn*. Left is ventral (V) and right is the dorsal (D) side. Compared with *foxQ2* expression, the expression of *hbn* is ‘shifted’ towards the dorsal side of AP. *nodal* is the ventral marker. Arrows in (E, F) indicate *hbn* expression in the stomodeum. Dot-lined circle in (K’) indicates the AP region. Serotonergic neurons in control embryos (L, M) are missing in Hbn morphants (N, O). (P, Q) Serotonergic neurons are not differentiated in the Δcad and Hbn-MO co-injected embryos. Hbn is an upstream gene of *tph*, *tryptophan 5-hydroxylase* (R, S), *zfhx* (T, U), and *fez* (V, W). Arrowheads in (R, T, V) indicate the precursors of serotonergic neurons. Arrows in (U) indicate lateral ganglion neurons.

In adding to FoxQ2, we focused on the function of Hbn, another AP-specific factor. *hbn* is initially expressed throughout the AP (Fig 2A), as previously described in different species [18]. During subsequent developmental stages, the expression of *hbn* progressively fades from the ventral half of the AP and appears at the dorsal/lateral ectoderm, adjacent to the AP, in the early gastrula stage (24 h) (Fig 2B and 2C). At the late gastrula stage (30 h), its expression is restricted to the dorsal/lateral ectoderm and it completely disappears from the anterior end of the AP (Fig 2D). In addition to its dorsal/lateral ectodermal expression, *hbn* appears at the upper lip region in the prism stage (36 h, Fig 2E, arrow), where it remains, at least until the pluteus stage (48 h Fig 2F, arrow). To compare the expression pattern of *hbn* with that of *foxQ2* or *tryptophan 5-hydroxylase* (*tph*), a serotonin synthase gene, we employed two-color fluorescence *in situ* hybridization. *foxQ2* and *hbn* expression nearly overlapped in the AP region of the unhatched blastula (Fig 2G), but *hbn* gradually faded from the region and a portion of the dorsal/lateral ectoderm that was adjacent to the AP began to express *hbn*, which resulted in the expression pattern of *hbn* being ‘shifted’ toward the dorsal side away from the AP (Fig 2H and 2I). By the late gastrula stage, *hbn* expression had completely disappeared from the *foxQ2* area (Fig 2J). Double staining of *hbn* and *tph* in the pluteus stage showed that the serotonergic neurons were differentiated at the edge of the AP, which was adjacent to the *hbn*-expressing region (Fig 2K and 2K’).

Hbn morphants developed into pluteus larvae without detectable defects in morphology or developmental timing, except for a defect in the elongation of the anterolateral arms at 72 h (*cf*. Fig 2N with 2L) and at 96 h (*cf*. S1C with S1A Fig). In addition, Hbn morphants have significantly fewer serotonergic neurons than normal embryos while non-serotonergic neurons at the AP and ciliary band are almost normal (Fig 2M, 2O, S1A–S1D and S1G Fig). Because the development of serotonergic neurons is affected by several signals from outside of the AP [9], we employed a Δcad-injected embryo to accentuate Hbn function under conditions where all other known signals were eliminated [22,23]. A Δcad-injected embryo, in which the initially fated AP contains a greatly increased number of serotonergic neurons (Fig 2P), is an appropriate system to analyze the intrinsic function of genes that are expressed within it, as was previously reported [6]. When Hbn was knocked down in Δcad-injected embryos, the development of serotonergic neurons was strongly inhibited, as was observed in normal morphants (Fig 2P and 2Q). These phenotypes were specific because they were also obtained when using a second morpholino (Hbn-MO2) that targeted a non-overlapping sequence in the mRNA (S1E and S1F Fig), and the microinjection of an mRNA encoding Hbn protein partially rescued the morpholino knockdown effect under the early cWnt-deficient condition (S2A–S2J Fig). These results indicate that Hbn is required for the development of serotonergic neurons in the AP.

To identify the step in which Hbn is involved during the development of serotonergic neurons, we examined Hbn morphants for the expression of *tph*, which is a terminal differentiation marker, and *zinc finger homeobox 1* (*zfhx1*; [8]) and *forebrain embryonic zinc finger* (*fez*), which are early neural markers [14]. In Hbn morphants, *tph* was not expressed in the neuroectoderm (Fig 2R and 2S, arrowheads show *tph*-cells in the control), which indicated that the transcription of *tph* required Hbn function. *zfhx1* and *fez* are downstream of FoxQ2 but are independent of each other. Hbn morphants expressed neither of these genes in the AP (Fig 2T–2W, arrowheads show the expression patterns of each gene in the control embryos). As expected, *zfhx1* expression in the lateral ganglion was not affected in Hbn morphants (Fig 2U, arrows). In adding to the previous report, which showed that the entire AP region had the potential to produce serotonergic neurons [8], these data indicated that Hbn is required for the specification of serotonergic neurons.

### TGF-ß signals regulate the dorsoventral patterning of *foxQ2* and *hbn*

**Fig 3:**
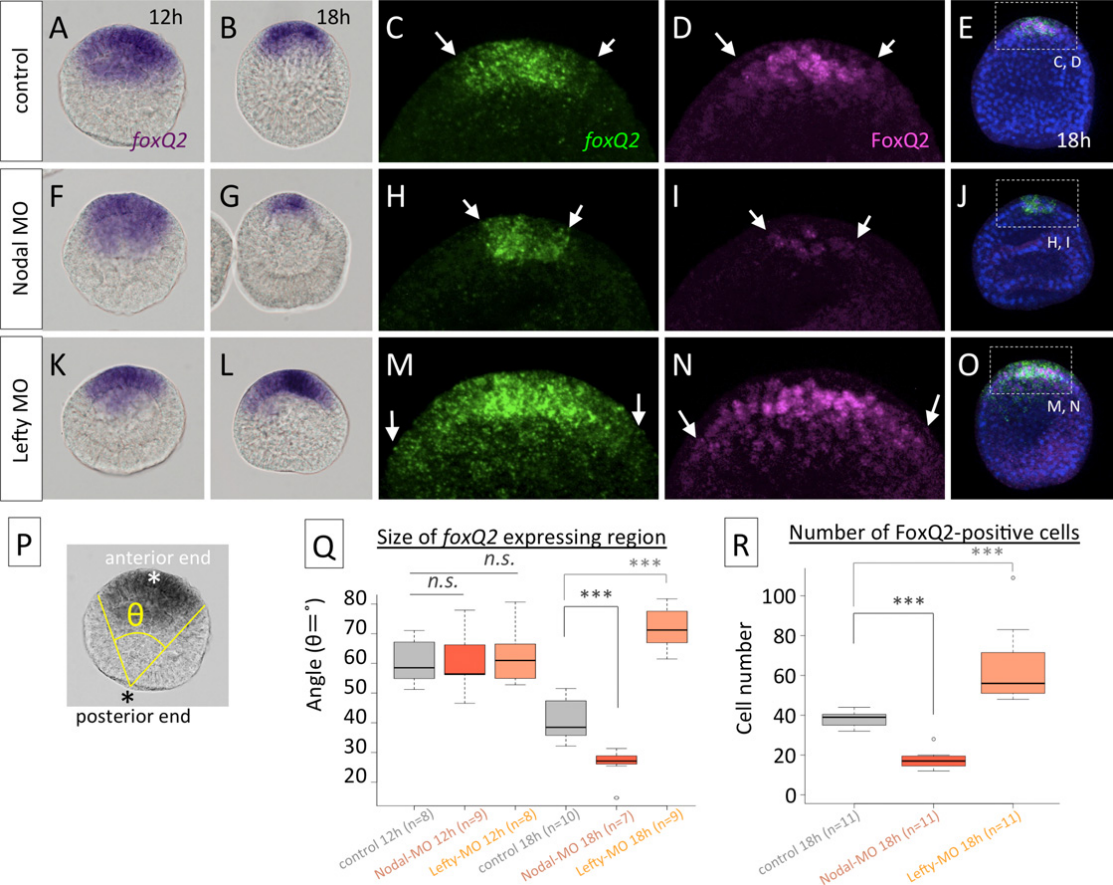
Nodal maintains *foxQ2* expression. *foxQ2* mRNA and FoxQ2 protein patterns in control embryos (A-E), Nodal morphants (F-J) and Lefty morphants (K-O). Arrows in (C, H, M) and (D, I, N) indicate the edge of the *foxQ2*-mRNA and FoxQ2-protein region, respectively. (P) The image shows how the *foxQ2*-expressing area was measured with the angle (θ**)** from the posterior end. (Q) Quantification of the size of the *foxQ2* region in the control (A, B), Nodal morphants (F, G) and Lefty morophants (K, L). The *foxQ2* region in Nodal morphants and Lefty morphants is significantly narrower and wider, respectively, than that in the control at 18 h, whereas they are all similar at 12 h. (R) Quantification of the number of FoxQ2-protein positive cells in the control (E), Nodal morphants (J) and Lefty morphants (O). *n*.*s*. not significant, ***P<0.001, Student’s *t*-test. *foxQ2* expression pattern is almost asymmetrical during all stages shown in this figure.

The change in the *foxQ2* expression pattern along the dorsoventral axis suggested that its expression may be regulated by or depend upon TGF-ß signals because cell fate specification along the secondary, dorsal-ventral axis of sea urchin embryos was determined by TGF-ß family members such as Nodal and BMP2/4 [23–25]. Therefore, we examined whether the Nodal pathway is involved in the regulation of *foxQ2* expression throughout development. In Nodal morphants, the size of the *foxQ2* region was smaller than that of control embryos at the hatched blastula stage (18 h) (*cf*. Fig 3G with 3B, quantification of *foxQ2* region was shown in P, Q), but they were invariant in unhatched blastulae (12 h) (Fig 3A, 3F and 3Q). The protein localization of FoxQ2 in hatched blastula also showed the same size as its mRNA pattern, and the immunochemical signal in Nodal morphants was weak (*cf*. Fig 3H–3J with 3C–3E, between arrowheads), which indicated that Nodal is required for maintaining *foxQ2* expression during the blastula stages. In contrast, in Lefty morphants, in which Nodal proteins are located throughout the ectoderm [26], the size of the AP in the hatched blastula stage was significantly wider than that in control embryos (Fig 3L–3O and 3Q), which indicated that misexpressed Nodal interferes with the restriction of the neuroectoderm during blastula stages. The difference in the *foxQ2*-mRNA positive region in controls, Nodal morphants and Lefty morphants measured with the angle from posterior pole was supported by the data, in which we counted the number of FoxQ2-protein positive cells in 18 h stages (Fig 3R). Based on these data, Nodal maintains the expression of *foxQ2* during blastula stages, and this mimics the process that occurs on the ventral side of the AP during normal development.

**Fig 4:**
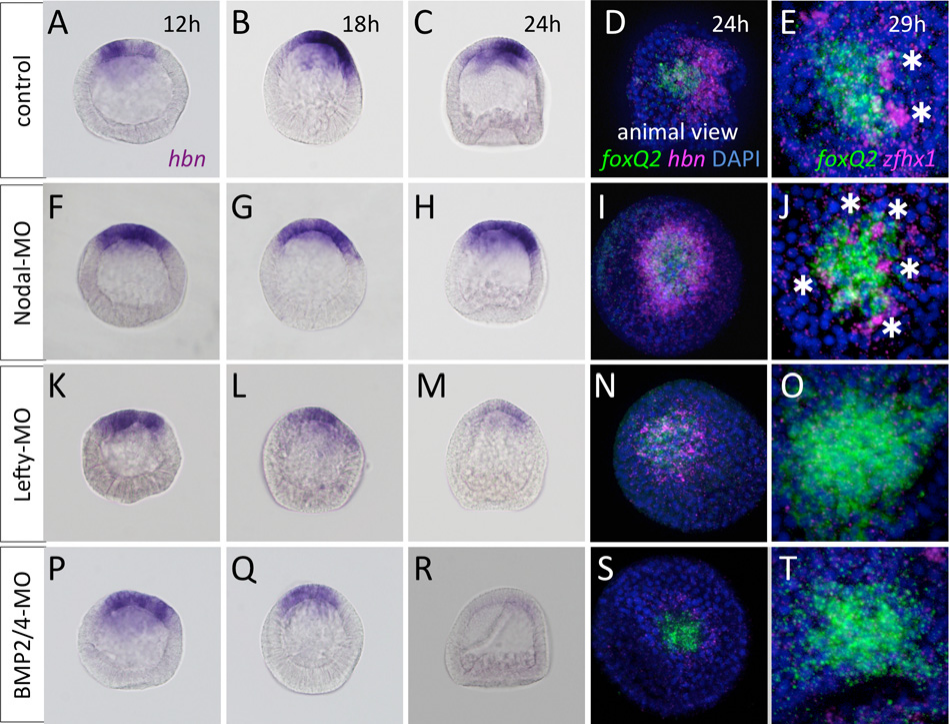
TGF-ß signals regulate the dorsoventral pattern of *hbn*. (A-E) *hbn* is shifted toward the dorsal side, where serotonergic neurons are differentiated (E) in control embryos. (F-J) *hbn* surrounds the *foxQ2* region and the serotonergic neurons are differentiated at the same region (J). Clear shifting toward the edge of the AP and strongly localized expression at later stages is not observed in Lefty morphants (K-O) or BMP2/4 morphants (P-T). Both have no serotonergic neurons. Asterisks indicate the precursors of serotonergic neurons, which express *zfhx1*.

Next, to investigate what controls *hbn* expression along the secondary axis, we observed its pattern in the embryos, in which the TGF-ß signals responsible for secondary axis formation were disturbed [23,25]. In Nodal morphants, *hbn* expression was shifted uniformly to the AP-adjacent region by the early gastrula stage (24 h) (*cf*. Fig 4H, 4I with 4C, 4D), which suggests that Nodal suppresses the expression of *hbn* on the ventral side of normal embryos. However, quantitative PCR (qPCR) data indicated that the amount of *hbn* mRNA was not significantly changed in the morphants or even in Nodal-misexpressed embryos (S3 Fig), suggesting that the function of a strong *hbn* inducer was missing in both Nodal morphants and misexpressed embryos. As a result of the uniform shifting of *hbn*, *zfhx1*-positive cells were distributed around the *foxQ2* region in Nodal morphants (Fig 4I and 4J, asterisks), whereas in normal embryos, the precursor cells of serotonergic neurons were present at the dorsal edge of the AP (Fig 4D and 4E). In Lefty or BMP2/4 morphants, in which the dorsal ectoderm is missing and the ventral ectoderm surrounds the AP [24,26], but in which *hbn* expression at the unhatched blastula stage (12 h) is not changed (*cf*. Fig 4K, 4P with 4A), the expression patterns became obscure after hatching and never showed clear shifting towards the edge of the AP region, unlike in control or Nodal morphants (Fig 4K–4N and 4P–4S). These results suggest that Lefty and BMP2/4 are required for maintaining the strong expression of *hbn* after the hatched blastula stage. In fact, because the significant decrease of *hbn* mRNA was observed only in BMP2/4 morphants by qPCR (S3D Fig), we concluded that BMP2/4 is essential for the maintenance of *hbn* on the dorsal side. Furthermore, when the clear shifting of *hbn* expression towards the edge of the AP was almost entirely missing in these morphants, no *zfhx1* expression was observed at the AP region (Fig 4N, 4O, 4S and 4T), resulting in the loss of all serotonergic neurons, as previously reported [27]. Taken together, the findings show that Hbn plays a role in an intrinsic system that determines the initial neural fate at the dorsal/lateral edge of the AP and that its expression patterns are highly regulated by TGF-ß signals along the dorsoventral axis.

### Canonical Wnt pathways are required for the disappearance FoxQ2 from the neurogenic AP, with precise timing

**Fig 5:**
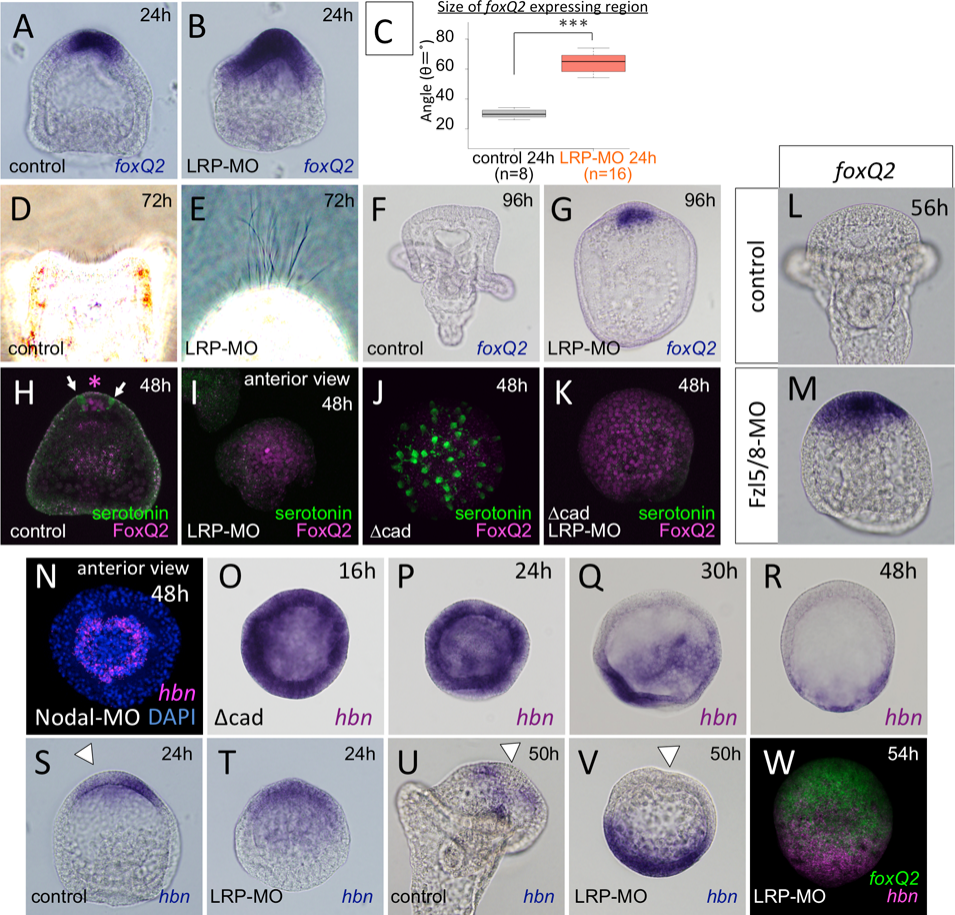
Canonical Wnt signal regulates the patterning of anterior neuroectoderm. In contrast to control embryos (A), LRP6 morphant had a wider *foxQ2* region (B). (C) Quantification of the size of the *foxQ2* region in control (A) and LRP6 morphants (B). ***P<0.001, Student’s *t*-test. LRP6 morphants retain the apical tuft at 72 h (E) and *foxQ2* (G) at 96 h, whereas both are diminished in control embryos at those times (D, F). The differentiation of serotonergic neurons is delayed and not observed at 48 h in LRP6 morphants (H, I). Arrows in (H) indicate serotonergic neurons. Asterisk shows *foxQ2* protein. This delay is independent of the presence or absence of Δcad mRNA injection (J, K). (L, M) *foxQ2* is maintained in Fz5/8 morphants. (N) *hbn* pattern in Nodal morphants. (O-R) Spatiotemporal expression pattern of *hbn* in Δcad-injected embryos. *hbn* mRNA is maintained at the anterior end until at least 24 h. The clearance occurs without early cWnt and TGF-ß signals even though the timing is delayed. *hbn* clearance is not observed in LRP6 morphant at 24 hr (S, T), but it occur by 50 h (U, V). (W) Double fluorescent *in situ* hybridization with *hbn* and *foxQ2* to confirm *hbn* clearance from the anterior end. White arrowheads indicate the location of *hbn* clearance.

The next question was what further restricts the *foxQ2* region further to the anterior end without Nodal signaling (Fig 3G and 3H). To investigate this question, we focused on the cWnt pathway because the inhibition of the early cWnt pathway interfered not only with AP restriction but also with AP patterning, resulting in the serotonergic neurons being differentiated in a dispersed manner (Fig 1A). Because the effect of an exogenous cadherin fragment (Δcad) on blocking cWnt [22] might be short-lived due to the short half-life of the injected mRNA, we blocked the function of low-density lipoprotein receptor-related protein 6 (LRP6: LC064120 for *H*. *pulcherrimus* LRP6), a co-receptor that acts with the Wnt receptor Frizzled (Fzl) to mediate the cWnt pathway. Based on previous reports, *LRP6* mRNA is expressed maternally, lasts throughout embryogenesis [28] and is present in all cells during the early stages [29]. The same observations were made in *H*. *pulcherrimus* embryos (S4A–S4F Fig). Although the localization of *LRP6* mRNA was uniform in embryos, the protein was missing in the ingressed and future mesodermal region at the mesenchyme blastula stage (S4G and S4H Fig). In LRP6 morphants, the protein-detection level was significantly decreased (S4I and S4J Fig; each image was captured in the same microscopic condition), and mesenchyme ingression was normal but no endoderm invagination was observed (Fig 5A and 5B). This result occurred because the mRNA and likely, protein of LRP6 were present maternally, and LRP6-MO could not block the early cWnt, unlike Δcad injection. In LRP6 morphants, the *foxQ2* expressing region was significantly wider than that of control embryos at 24 h (Fig 5A–5C), and the dorsal-ventral polarity in the ectoderm was normal based on *nodal* and *hnf6* expression patterns (S4K–S4O Fig; [23]). The morphant retained the apical tuft, which should disappear by 72 h during normal development (Fig 5D and 5E; [13]), and, intriguingly, its essential regulatory gene, *foxQ2*, was also still detected at 96 h (Fig 5F and 5G). This result indicates that LRP6-mediated cWnt signaling is required for suppressing *foxQ2* expression in the AP.

Despite the wider *foxQ2* region, LRP6 morphants had no differentiated serotonergic neurons at 48 h (Fig 5H and 5I). This result was likely derived from the maintenance of FoxQ2 in each cell in the expanded AP. This was confirmed in 48 h Δcad-embryos and Δcad-LRP morphants because a number of serotonergic neurons and no FoxQ2 protein were observed in the former, while strong FoxQ2 signal and no serotonergic neurons were observed in the latter (Fig 5J and 5K). Taken together, these results indicate that an LRP6-mediated signal seems to be involved in the disappearance of FoxQ2 from the AP and for the precise differentiation timing of serotonergic neurons.

Among the Frizzled receptors for the Wnt pathway in sea urchin embryos, it was reported that Fzl1/2/7 and Fzl5/8 are expressed in the ectoderm [30], and Fzl5/8 is likely the only Fzl receptor whose function we can analyze during the modification of the restricted AP region because Fzl1/2/7 morphants lose the entire AP from the very beginning of its formation [31]. Thus, we investigated whether Fzl5/8 mediates the LRP6-based cWnt pathway that controls the disappearance of FoxQ2 from AP cells. In Fzl5/8 morphants at 56 h, the expression of *foxQ2* was maintained (Fig 5M), whereas control embryos lost the *foxQ2* message at this stage (Fig 5L). This result suggested that Fzl5/8 functions in controlling the cWnt at the AP region.

The disappearance of *hbn* from the anterior end occurred normally in Nodal morphants, and the *hbn*-expressing region surrounded the central part of the AP (Fig 5N), which suggests that the disappearance of *hbn* expression is independent of the dorsoventral axis formation by TGF-ß signals (Fig 4). To confirm this finding and to investigate the involvement of the cWnt pathway as an upstream factor of TGF-ß signals during *hbn* clearance, we employed Δcad-embryos, which lack all known zygotic signals including early cWnt and TGF-ß signals [22,23]. In these embryos, *hbn* is expressed throughout the entire region during the early stages (Fig 5O and 5P). Then, *hbn* disappears from the central portion of the AP by 30 h (Fig 5Q; [18]). The *hbn*-negative region is progressively expanded, and the *hbn*-expressing region is observed only in the squamous epithelia in the posterior half of 48 h Δcad-embryos (Fig 5R). This result supports previous data from a different species, *S*. *purpuratus* [18]. This disappearance pattern of *hbn* in Δcad-injected embryos was spatially similar to that observed in the anterior end area of normal embryos, which suggests that the spatial control of the disappearance of *hbn* expression from the anterior end of the AP is independent of cWnt and TGF-ß signals.

Focusing on the *hbn* expression pattern in Δcad-embryos in detail, we found that the disappearance of the gene from the anterior end was delayed. In normal embryos, *hbn* began to be diminished from the anterior end of the AP by 18 h (Fig 2H), but it did not disappear until 30 h in Δcad-embryos (Fig 5Q). In addition, because the cWnt pathway likely regulates the disappearance of *foxQ2* as mentioned above, we investigated the function of LRP6 on *hbn* regulation. *hbn* gene expression remained in the entire anterior half in LRP6 morphants at 24 h, at when the clearance of the gene was quite obvious in the controls (Fig 5S and 5T). However, *hbn* had disappeared from the AP by 50 h, as in normal embryos (Fig 5U and 5V). This was confirmed by the result from double fluorescent *in situ* hybridization for *foxQ2* (Fig 5W). In addition, the disappearance of *hbn* from the AP region is independent of the absence of LRP6 function (S4P Fig). Based on these observations, the LRP-mediated cWnt signal is not required for the disappearance of *hbn* from the AP, but it is required for the control of the timing of its clearance.

### Wnt7 functions as one of the ligands in the cWnt pathway at the neurogenic AP

**Fig 6:**
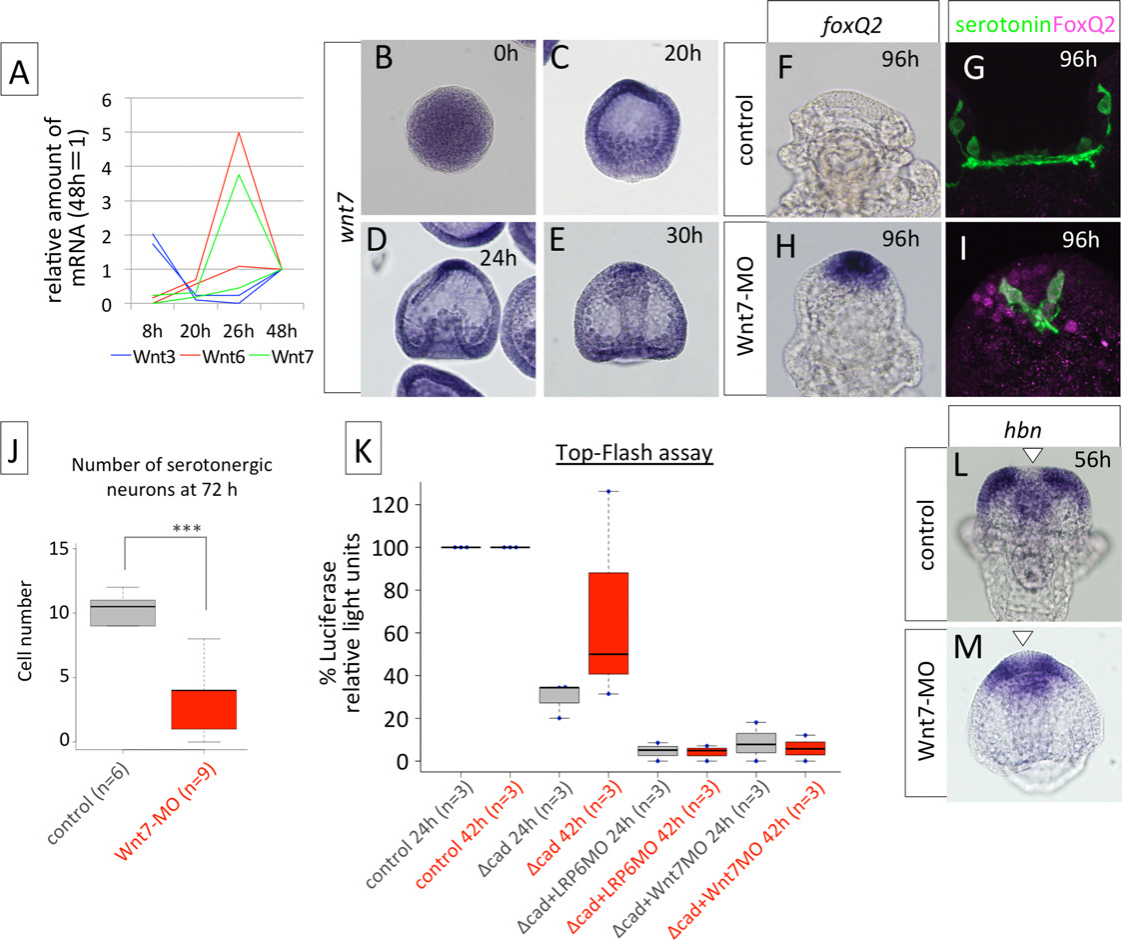
Wnt7 is a ligand for cWnt pathway that regulates the patterning of anterior neuroectoderm. (A) Quantitative PCR data revealing wnt3, wnt6 and wnt7 during sea urchin embryogenesis. The data from two independent batches are shown. (B-E) *wnt7* expression patterns during sea urchin embryogenesis. In the Wnt7 morphants, *foxQ2* mRNA (H) and protein (I) are remained until late stages, e.g., 96 h, whereas they are not expressed in the control (F, G). The number of serotonergic neurons is significantly smaller in Wnt7 morphants at 72 h (J) and 96 h (I) than in controls (G, J). ***P<0.001, Student’s *t*-test. (K) Top-Flash assay revealed that LRP6 and Wnt7 are involved in the canonical Wnt pathway at the AP region. (L, M) *hbn* clearance occurs in Wnt7 morphant, indicating that *hbn* is not a strong target of the cWnt pathway. White arrowheads indicate the location of *hbn* clearance.

To find the ligands for cWnt signaling in suppressing *foxQ2*, we focused on later-expressed Wnts in this study because the early Wnts that function in endomesoderm formation might have indirect effects on AP regulation. Based on the temporal expression profile previously reported, Wnt3 (LC064118 for *H*. *pulcherrimus* Wnt3), Wnt6 (LC0641198 for *H*. *pulcherrimus* Wnt6) and Wnt7 (LC064119 for *H*. *pulcherrimus* Wnt7) are expressed relatively late [28,32]. In *H*. *pulcherrimus*, *wnt3* is expressed during the cleavage stage but not after the blastula stage, according to qPCR, whereas *wnt6* and *wnt7* were expressed after hatching (Fig 6A). Based on perturbation experiments, it is suggested that Wnt7 functions as a ligand for the cWnt pathway, and Wnt6 for non-cWnt pathways, and those reasons will be explained in this section for Wnt7 and in the next section for Wnt6. *wnt7* was expressed broadly at 20 h and was abundantly expressed in the AP. The broad expression of *wnt7* and its strong expression in the AP were invariable until 30 h (Fig 6B–6E).

In Wnt7 morphants, *foxQ2* mRNA was still expressed in the AP region even at 96 h (Fig 6F and 6H) and FoxQ2 protein remained to be detected in AP cell nuclei at the same stage (Fig 6G and 6I). Because FoxQ2 persisted, the differentiation of serotonergic neurons was extremely delayed. The number of serotonergic neurons at 72 h was significantly smaller than that in controls (Fig 6J). Because FoxQ2 persistence and missing serotonergic neurons were similar characteristics observed in LRP6 morphants, these results suggest that Wnt7 functions as a ligand of the cWnt pathway in mediating the differentiation of serotonergic neurons through the suppression of FoxQ2 expression.

Although our data so far have suggested that the cWnt pathway is involved in AP patterning through suppressing *foxQ2* expression, nuclear ß-catenin was not observed in the region until at least the 8^th^ cleavage stage [22]. Because the antibody that recognizes nuclear ß-catenin in *H*. *pulcherrimus* is not available, we performed a TCF-luciferase reporter system (Top-Flash) assay to measure the level of cWnt signal [33,34]. Δcad-injected embryos have only approximately 30% Top-Flash activity compared to controls at 24 h (Fig 6K). This decreased activity tends to recover as the embryos grow due to the degradation of exogenous Δcad-mRNA and/or protein. However, without LRP6 or Wnt7 functions, Top-Flash activity remains low, and the scores of the activity are significantly lower than those in controls at both 24 h and 42 h (Fig 6K). These data support that in the AP region cWnt functions through the Wnt7-LRP6 pathway in suppressing *foxQ2* expression. In contrast, Wnt7 morphants had normal clearance of *hbn* expression from the anterior end (Fig 6L and 6M), supporting the idea that cWnt is not involved in controlling *hbn* expression patterns.

Because one of the interesting questions in this study is that of which signaling pathway crosstalks with Nodal signaling during the regulation of AP patterning (Fig 3), a cWnt pathway regulated by Wnt7 might be the candidate. In fact, when the Wnt7 function was blocked, the *foxQ2* region was wider than in normal embryos at the blastula stage (S5A, S5B and S5D Fig). The excessive restriction of the *foxQ2* region that was observed in Nodal morphants (S5A and S5C Fig) was rescued in Nodal-Wnt7 double morphants (S5A and S5E Fig), suggesting that Wnt7 is the factor that restricts *foxQ2* expression to the anterior end and that the Nodal signal inhibits a Wnt7-mediated signaling pathway during the blastula stages.

### Wnt6-mediated non-cWnt pathway is required for *hbn* disappearance from the anterior end of the AP

**Fig 7:**
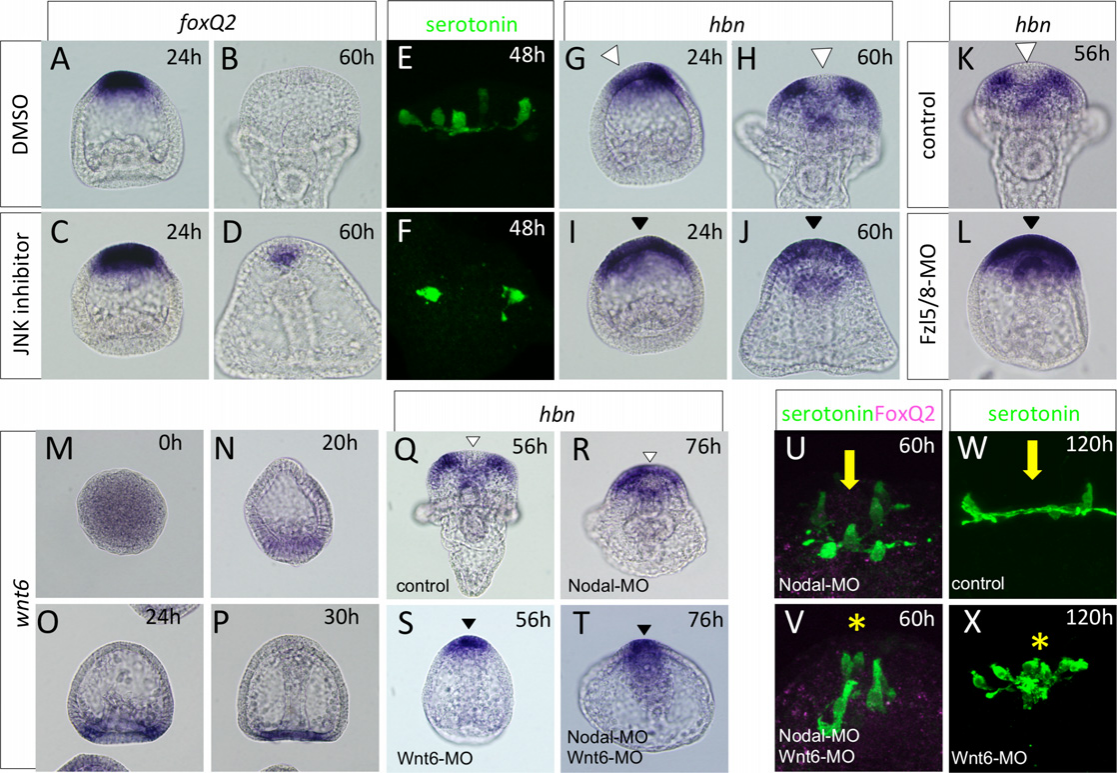
Wnt6 is a ligand for non-cWnt pathway that regulates the patterning of *hbn* expression. (A, C) *foxQ2* expression in the control (DMSO-treated) and JNK inhibitor treated embryos at 24 h. (B, D) *foxQ2* expression in the control (B) and in JNK inhibitor treated embryo (D) at 60 h. A trace level of *foxQ2* was expressed in JNK inhibitor treated embryos, whereas *foxQ2* is completely diminished in the control at this stage. (E, F) The serotonergic neurons are differentiated at 48 h with or without JNK function. The AP region is magnified. (G-J) *hbn* clearance does not occur in JNK inhibitor-treated embryos. (K, L) *hbn* remains at the anterior end of AP in Fzl5/8 morphants at 56 h. (M-P) *wnt6* was strongly expressed in the veg2 endoderm region at 20 h, and afterward it was maintained in the vegetal plate until at least 30 h. (Q, S) *hbn* does not disappear from the anterior end and is not expressed in the non-AP region in Wnt6 morphants, and this is independent of the presence or absence of Nodal (R, T). (U-X) Without Wnt6 function, serotonergic neurons are differentiated at the anterior end of the AP. To visualize the phenotype more clearly during early stages, Nodal-MO was simultaneously injected. Arrow in (U, W) indicates the anterior end that misses serotonergic neurons. Asterisk in (V, X) shows that the serotonergic neurons are present at the central part of AP. White and black arrowheads indicate the location of *hbn* absence and presence, respectively.

We next focused on the function of non-cWnt signals on AP patterning. As it is downstream of early cWnt signals from the posterior side, a c-Jun N-terminal kinase (JNK) signal functions in the restriction of the AP to the anterior end [31]. To examine whether a JNK signal also plays a role in AP patterning, we applied a JNK inhibitor from 2–4 cell stages and analyzed the expression patterns of *foxQ2* at the desired stages. As was previously reported, the restriction of *foxQ2* to the anterior end was inhibited in the absence of JNK function at 24 h (Fig 7A and 7C). *foxQ2* disappearance from the AP was delayed in JNK-inhibited embryos, but the remaining signal was weak, and its area was very small at 60 h (Fig 7B and 7D). Unlike the cWnt pathway, the JNK pathway seem to be weakly involved in the maintenance of *foxQ2* expression because the timing of the initial differentiation of the serotonergic neurons was slightly delayed (48 h, Fig 7E and 7F), but a number of serotonergic neurons were differentiated in the expanded AP one day later [31]. We next focused on the function of non-cWnt signals on *hbn* expression, and used a JNK inhibitor and analyzed the expression patterns of *hbn*. *hbn* clearance from the anterior end at 24 h was not observed in JNK-inhibited embryos (Fig 7G and 7I, arrowheads). In addition, the clearance was intriguingly not observed in JNK-inhibited embryos, even in the later stages (*cf*. Fig 7J with 7H; arrowheads). Together, these results suggest that a JNK signal acts as a part of non-cWnt signaling and that it mainly plays a role in the clearance of *hbn* from the anterior end of the AP.

As mentioned above, Fzl5/8 is likely the only Fzl receptor whose function we can analyze during the modification of the restricted AP region [31]. Here, we investigated whether Fzl5/8 mediates the non-cWnt pathway that controls the clearance of *hbn* from the central part of the AP. In Fzl5/8 morphants at 56 h, the expression of *hbn* was maintained (Fig 7L) whereas all control embryos had no *hbn* mRNA (Fig 7K). This result suggested that Fzl5/8 mediates the non-cWnt signal at the AP region.

To investigate the ligands for non-cWnt signaling in *hbn* patterning, we focused on Wnt6 because Wnt7 was not involved in the regulation of *hbn* expression (Fig 6). *wnt6* is expressed in the veg2 endoderm region and is not obvious at the ectoderm (Fig 7M–7P). The morphology of Wnt6 morphants did not resemble a normal pluteus stage even at 56 h and had a straight archenteron and no pluteus arms (Fig 7Q and 7S). Focusing on the *hbn* expression pattern, we found that it did not disappear from the central part of the AP region in Wnt6 morphants (Fig 7S). This result was confirmed by experiments in embryos that were doubly injected with Δcad and Wnt6-MO, in which *hbn* was broadly expressed in the expanded AP (S6I and S6K Fig). To clarify this finding, we employed Nodal morphants; without Nodal function, *hbn* “shifting” to the periphery of the AP is more obvious (Fig 7R). The morphants, in which Nodal and Wnt6 were simultaneously knocked down, showed no *hbn* disappearance from the central part of the AP (Fig 7T). This result clearly indicated that Wnt6 is required for *hbn* suppression in the AP. Because *hbn* was maintained in the AP in Wnt6 morphants, serotonergic neurons were differentiated not at the edge of the region but at the anterior end of the AP (*cf*. Fig 7V, asterisk with U, arrow). These data are supported by the serotonergic neural patterns in later stage, in which the differentiated serotonergic neurons gather at the anterior end in Wnt6 morphants even if there is no Nodal inhibition (Fig 7W and 7X). In addition, the nuclear localization of FoxQ2 had already disappeared at 60 h, as in normal embryos (Fig 7U and 7V), indicating that Wnt6 is not strongly involved in the regulation of the FoxQ2 expression pattern, which is mediated by LRP6/cWnt signaling. Although it is difficult to distinguish, these results suggest that Wnt6 functions as one of the players in the non-cWnt pathways that regulate AP patterning, especially in the control of *hbn* expression.

Because the expression patterns of *foxQ2* and *hbn* are complementary after the gastrula stage in normal embryos, FoxQ2 is another candidate for suppressing *hbn* expression. To examine this possibility, we investigated *hbn* expression patterns in FoxQ2 morphants. The disappearance of *hbn* occurred normally in the morphants (S7A–S7F Fig), which suggests that FoxQ2 and its downstream genes do not regulate the suppression of *hbn* expression at the AP in later embryos. Furthermore, in Hbn morphants, the expression pattern of *foxQ2* was the same as that in normal embryos (S7G–S7J Fig), indicating that FoxQ2 and Hbn are mutually independent.

## Discussion

**Fig 8:**
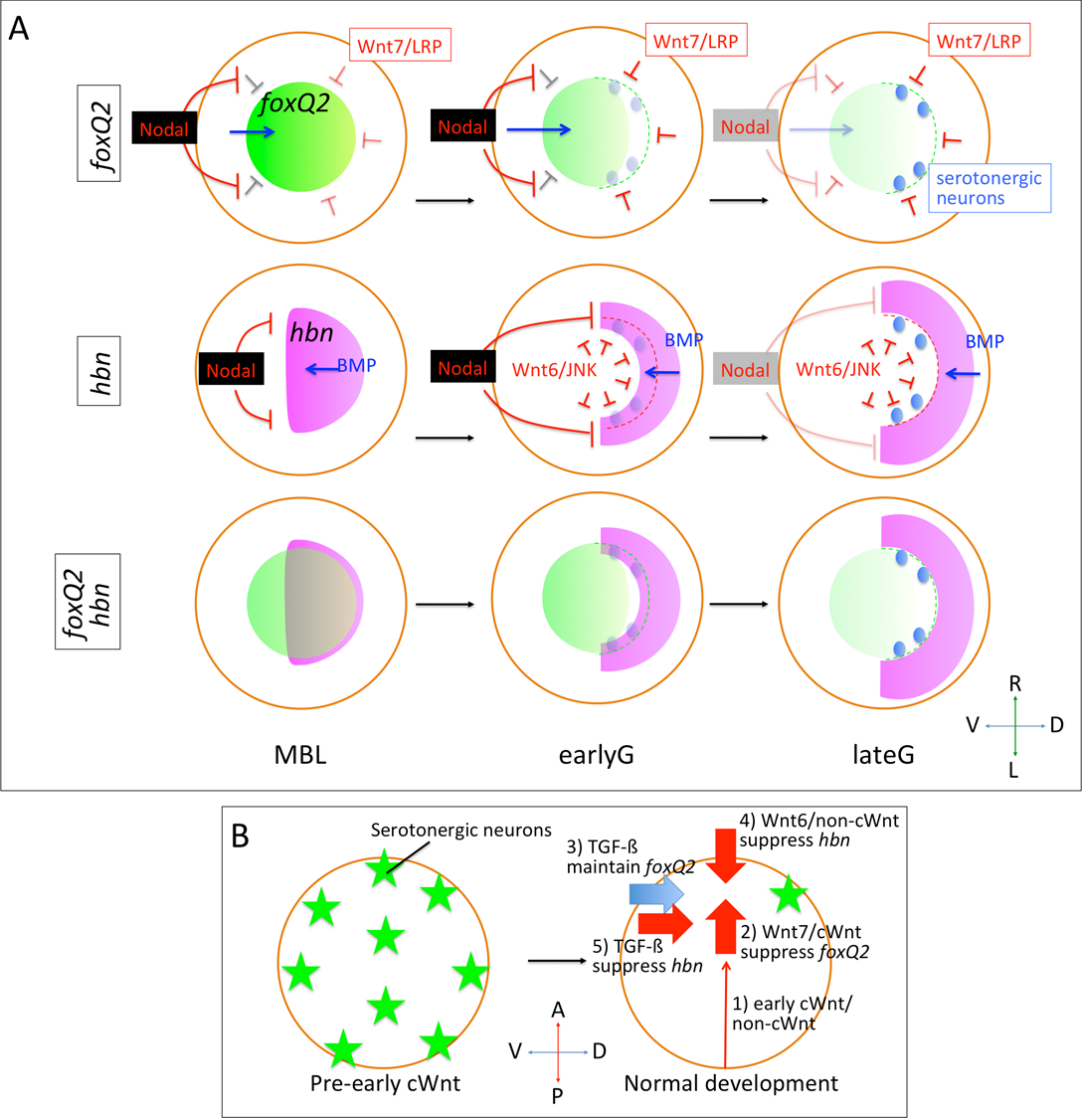
Models for anterior neuroectoderm patterning. (A) Schematic images of signaling pathways regulating *foxQ2* and *hbn* expression. Anterior views. *foxQ2* (green) is suppressed by cWnt mediated by Wnt7/LRP6 from the blastula to gastrula stages. Nodal inhibits the signal from the ventral side. Serotonergic neurons (blue) begin to be differentiated in the *foxQ2*-missing area, i.e., the dorsal/lateral edge of the AP. *hbn* (magenta) expression shifts towards the dorsal/lateral edge of the AP. Nodal and non-cWnt mediated by Wnt6/JNK signals regulate the suppression, whereas BMP2/4 promotes its expression. The dotted line indicates the dorsal border of the AP. MBL, mesenchyme blastula; early G, early gastrula; late G, late gastrula. (B) Summary of signaling pathways regulating the serotonergic neural fate at the dorsal/lateral edge of the AP. Before receiving extrinsic signals beginning from early cWnt, the cell fate of most cells in sea urchin embryos are the AP, which differentiates a number of serotonergic neurons. However, under normal conditions, five signals restrict its fate to a small region.

Here, we reveal the molecular mechanisms that control the patterning of the anteriorly restricted neurogenic AP ectoderm along the anteroposterior and dorsoventral axes in the sea urchin embryo, i.e., how embryos pattern the initial neuroectoderm to let the specific neurons differentiate only at the correct location (Fig 8). Although a number of genes that are expressed at the neurogenic ectoderm were identified during the genome sequencing project of *S*. *purpuratus* [17,18,35], those analyses were not sufficient to explain the molecular pathways that regulate AP formation and neural differentiation. Our data show that the expression of transcription factors inside AP must be precisely controlled by the intrinsic and/or extrinsic TGF-ß and Wnt molecules and that this regulation is essential for the development of AP and neurons. We also reveal the function of Hbn in specifying the initial neuroectodermal fate. To our knowledge, this is the first study revealing the molecular function of Hbn in any animals, although the expression pattern of *hbn* has been reported in several species. With Hbn function, we must consider the function of FoxQ2 in the specification and differentiation of neurogenic AP. FoxQ2 is initially required for the specification of most of cell types in the AP by the mesenchyme blastula stage [12], but it is not required later for neural differentiation because its function is to maintain apical tuft gene expression [13]. Therefore, the key to understanding the molecular mechanisms that maintain and suppress the initial neuroectodermal fate is the regulation that controls these two transcription factors in sea urchin embryos.

### TGF-ß signals regulate the dorsoventral patterning of the neurogenic ectoderm

It was previously reported that serotonergic neurons in the neurogenic AP of the sea urchin embryo are formed at the dorsal/lateral edges of the region [9,10] and that the differentiation of serotonergic neurons at the ventral side is suppressed by Nodal, which is expressed in the ventral ectoderm [6]. In this study, we revealed that *hbn* expression is suppressed by Nodal on the ventral side but maintained by BMP2/4 on the dorsal side. *hbn* expression is eliminated from the animal pole, likely by the non-cWnt pathway mediated by Wnt6/JNK after the blastula stage, which will be discussed below, and its pattern forms a horseshoe-like shape (Fig 8A). This pattern was not reported in another sea urchin, *S*. *purpuratus* [18], but, in *H*. *pulcherrimus*, it is clear that the expression is missing at the ventral side of the normal AP. The loss of Nodal function supports this observation because Nodal morphants have a ring-like shape of *hbn* expression around the neurogenic ectoderm (Fig 4). Because the expression of *foxQ2* is also under the control of secondary axis formation by the Nodal and BMP2/4 pathways (this study; [14]), we need to know whether Nodal and/BMP2/4 regulation is direct or indirect by further experiments, including chromatin immunoprecipitation analysis aimed at uncovering the cis-regulatory modules of *foxQ2* and *hbn*.

In vertebrates, TGF-ß signaling also functions in the neural plate patterning along the dorsoventral body axis [36]. For example, *bmp2* and *bmp7* expressing outside of the neural plate are necessary for the development of noradrenergic neurons through the induction of the homeodomain protein, *phox2a*, in zebrafish embryos [37]. Nodal, on the other hand, is required for suppressing the precocious acquisition of forebrain characteristics in mouse embryos [38]. Our data indicated that sea urchin embryos use the similar mechanisms to pattern the neuroectoderm, controlling the timing and location of the differentiation of serotonergic neurons. In addition, because there are other types of neurons present on the ventral side of the AP in sea urchin embryos [7], future investigations regarding the relationship between in-cell factors characterizing those neurons and TGF-ß signaling coming from the outside of the neurogenic AP region will lead us to understand the conserved mechanisms of neural patterning throughout the animal kingdom.

### Canonical and non-canonical Wnt signals regulate the anteroposterior patterning of the neurogenic ectoderm

Persistent FoxQ2 and apical tufts in the AP region in LRP6 morphants strongly indicate that the cWnt pathway is required to suppress FoxQ2 and exert a neural fate, although it has been reported that early cWnt, visualized with the nuclear localization of ß-catenin, was observed only at the posterior half of the embryo until the gastrula stage [22]. In addition, our results suggest that Wnt7 works as a ligand in the LRP-cWnt pathway in the AP that suppresses FoxQ2 with precise timing (Fig 6). Although we could not rule out the possibility that Wnt7 functions indirectly from outside of the AP of normal embryos, based on its expression patterns, the strong expression of *wnt7* at the thickened AP of normal (Fig 6) and Δcad embryos (S6E and S6H Fig) suggested that it plays an intrinsic role in FoxQ2 suppression within the AP. The difference between LRP6 morphants (abundant FoxQ2 and no serotonergic neurons) and Δcad embryos (less FoxQ2 and a number of serotonergic neurons) might be attributed to the lifetime length of exogenous Δcad mRNA and/or protein. Because the injected mRNA can last approximately 24 hr (e.g., S8A and S8B Fig), only early cWnt but not later cWnt is suppressed in Δcad embryos. This idea was well supported by Top-Flash assay (Fig 6). Of course, we cannot completely rule out the possibility that Δcad alone is not sufficient to block all cWnt, even during the early stages. The future TOP-Flash assay during the early stages can answer this question. Because *foxQ2* expression is at a gradient from anterior tip to periphery (Fig 1; [11]), the area of the biggest effect of *foxQ2* removal by Wnt7/cWnt might be the edge of the AP region, resulting in serotonergic neurons starting the differentiation process at the position. A previous report [31] showed that *foxQ2* was expressed at the posterior half, where this gene is never detected by *in situ* hybridization in normal embryos, when Axin was misexpressed, and they implicated that *foxQ2* is originally expressed throughout the embryo and early cWnt at the posterior half suppressed it. Thus, it is expected that the mechanisms suppressing *foxQ2* expression were also applied in the AP region after it is restricted to the anterior end.

The results from JNK inhibition led us to consider that non-cWnt is involved in neurogenesis in the AP of the sea urchin embryos (Fig 7). Additionally, data regarding its ligand, Wnt6, supported our JNK result (Fig 7). However, it is still not clear how Wnt6 mediates non-cWnt signaling in the AP region. Because it has been reported that *wnt6* is zygotically expressed at the vegetal plate and functions in endomesoderm formation [30,39], it is possible that the signal is indirect. However, our data using Δcad and Wnt6-MO indicated that Wnt6 could function within the AP region even though the expression of mRNA at that location is faint (S6 Fig). More detailed studies of its distribution and function with protein level will be necessary to understand the complete mechanisms of Wnt6 function. In contrast to the Wnt7 data, the normal disappearance of FoxQ2 in Wnt6 morphants indicated that Wnt6 did not function as a ligand of the cWnt pathway in the AP. The non-cWnt includes pathways other than JNK, planar cell polarity (PCP) and Ca^2+^ pathways [40], suggesting that those pathways are also involved in AP patterning in the sea urchin embryo. In fact, as a ligand for the non-cWnt pathway, Wnt6 might not be sufficient because JNK inhibition led to some *foxQ2* remaining in the AP, indicating that the JNK pathway weakly acts in suppressing FoxQ2 and affects the precise control of the timing and the location of serotonergic neurons (Fig 7). It was reported that the JNK pathway functions in restricting the AP region, represented by *foxQ2* and *hbn* expression, to the anterior end during blastula stages [31], but the Wnt6 morphant had no expanded AP and tended to have a more restricted *hbn* region, supporting the idea that other ligands for non-cWnt signaling function during anterior neuroectoderm formation.

Our results suggest that cWnt and non-cWnt signaling function in repressing FoxQ2 and Hbn, respectively, but we cannot completely rule out the possibility that each pathway affects both types of repression. This is because LRP6 morphants showed slightly delayed *hbn* clearance and JNK inhibition allowed some *foxQ2* to remain in the AP region. Because cWnt and non-cWnt antagonize each other in some biological processes [41], this cross-reaction might be normal in AP formation in sea urchin embryos. We must also consider the functional combination of frizzled receptors, secreted frizzled-related protein, and Dickkopfs (Dkks) to determine the complete involvement of the Wnt pathways in AP patterning. In fact, the restriction of the AP to the anterior end is managed through their combination with other Wnt ligands, such as Wnt1 and Wnt8 [31], and the patterning of anterior structure, including neuroectoderm, is also regulated those factors in other deuterostomes [42–44]. In contrast, both LRP6 and Wnt7 morphants failed to finish the restriction of the AP region by the blastula stages (Figs 5 and 6), similar to Wnt1 and Wnt8 morphants [31], suggesting that those factors affect the early cWnt events that restrict AP to the anterior end. Our data using Fzl5/8 morphants suggested that this frizzled functions as a receptor for both cWnt and non-cWnt signals as was observed in other systems [45]. This relationship strongly supports the idea that cWnt and non-cWnt cross-react with each other through sharing the frizzled receptor during AP patterning, although we cannot rule out that other types of frizzled, which we did not analyze in this study, are more essential for each pathway. Adding to our knowledge of the involvement of these molecules, biochemical analyses to reveal ligand-receptor associations will be conducted in the future to understand the complete pathway regulating AP formation in the sea urchin embryo.

As mentioned in the Introduction, the anterior neural fate in vertebrates is restricted by Wnt signaling from the posterior side [5]. Posterior Wnt signals are also reported in invertebrates, such as sea urchins [31,46,47] and amphioxus [48,49]. These species commonly use Wnt signals to establish posterior identities during early development. In this study, we found that *wnt7* is expressed in the AP region and required for the differentiation of serotonergic neurons as a ligand for the cWnt pathway (Fig 6), indicating that the sea urchin embryos utilize cWnt signaling at both the posterior and anterior ends. In addition, anterior and posterior cWnt share a function, repressing *foxQ2* expression (in this study, [12,31]). The simple mechanism that cWnt suppresses *foxQ2* with shifting the functional timing, early at the posterior and later at the anterior ends, enables embryos to have a complicated body plan along the anterior-posterior body axis: FoxQ2 initially specifies the AP region only at the anterior end and later it disappears from the AP to let the serotonergic neurons differentiate within it. We accidentally found this both-end cWnt signaling because we blocked the early cWnt at the posterior end using exogenous mRNA encoding Δcad, which has a short life-time. If we permanently and completely inhibit some of the components of the cWnt pathway, it might be difficult to recognize the later functioning anterior cWnt. It is possible that this type of anterior cWnt commonly functions in other systems during early development because in vertebrates it was reported that the *wnt7* family was expressed in the developing anterior neuroectoderm region [50].

### Crosstalk between TGF-ß and Wnt signals during patterning of the neurogenic ectoderm

One of the most interesting findings in this study is that the dorsoventral Nodal pathway might interfere with the anteroposterior Wnt pathway during the embryogenesis of the sea urchin. Because a number of studies previously revealed the molecular mechanisms of cell fate specification along these embryonic axes in many species, we have now accumulated the information to determine, for example, how the anteroposterior axis is formed by the bicoid gradient in the fly [51–53] or how left-right asymmetry is created by Nodal flow in mice [54,55]. However, because embryos must control cell fate specification along all three body-axes in our three-dimensional world with precise timing, the formation of the body axes should not be independent of each other. Thus, the information from processes that occur along each axis should be integrated with a high degree of sophistication and affect cell-fate specification during each step of embryogenesis. We have previously reported that a single transcription factor links anteroposterior-dorsoventral axis formation in the sea urchin embryo and that it regulates the timing of the onset of specification of the secondary axis downstream of primary axis formation [12]. By combining our results with previous reports, we propose the following five combinational signaling steps that regulate serotonergic neuron formation at the dorsal/lateral edge of the AP in the sea urchin embryo (Fig 8A and 8B): 1) posterior cWnt/non-cWnt signaling restricts the AP, which is specified by early FoxQ2, to the anterior end [6,31], 2) Wnt7/cWnt suppresses late FoxQ2, which induces the apical tuft cilia and represses neural fate, at the edge of the restricted AP along the anterior-posterior axis, 3) dorsoventral Nodal suppresses Wnt7/cWnt to maintain late FoxQ2 at the ventral side, 4) Wnt6/non-cWnt suppresses the neural specifier Hbn, preventing its expression in the anterior end, and 5) BMP2/4 strongly maintains the expression of neural specifier Hbn at the dorsal side whereas Nodal suppresses it at the ventral side. After the AP is restricted, the regulation of the expression of two opposing functional transcription factors, the neural specifier Hbn and late neural suppressor FoxQ2, is accomplished by the molecular mechanisms of neural patterning in the AP. Crosstalk between Wnts along the primary axis and Nodal along the secondary axis is carried out during the process of suppressing the serotonergic neural fate at the ventral side of the AP. Although suppressing the specifier of neurons, Hbn, at the ventral side seems to be sufficient, maintaining the suppressor, FoxQ2, within the same region is a great supporting system for embryos to ensure the removal of the neural fate.

Our data suggested that the effect of Nodal suppressing cWnt at the AP region seems to reach to the dorsal edge (Fig 3). However, because it is reported that Nodal can diffuse to short range in AP region [56], we do not know how the Nodal pathway controls the sizing of the entire AP through interactions with cWnt signaling in the AP. As *wnt7* is expressed abundantly in the AP (Fig 6), the Nodal pathway might affect its expression regulation even though Nodal itself can bind the receptor in a few rows at the ventral edge of the AP. In addition, the downstream factors of Nodal signaling, e.g., Lefty and BMP2/4, can diffuse to the AP region [12,14,23], and they might interact with cWnt pathway directly or indirectly to regulate the size control of the AP.

In *S*. *purpuratus*, it was indirectly implicated that Nodal regulates the expression of *foxQ2* by controlling transcription factors, Not, and Emx. Their relationships seem to be complicated along the spatiotemporal patterning [57,58], and none of them have yet been analyzed in *H*. *pulcherrimus*. However, our data on Nodal loss-of-function and gain-of-function are quite reproducible during those stages (Fig 3), and even in *S*. *purpuratus* Nodal morphants had a smaller AP, judging from the distribution of serotonergic neurons [18], supporting the idea that Nodal maintains the *foxQ2* expression in the sea urchin embryos. The precise cis-regulatory analysis of the *foxQ2* expression pattern in the future will lead us to understand the detailed molecular mechanisms of how TGF-ß signals control AP patterning.

### Exerting neural fate through FoxQ2 and Hbn functions

Because we have not yet succeeded in completely dissociating single cell-cultures, we cannot, strictly speaking, conclude that the default fate of sea urchin cells is the neuroectoderm or neurons. However, if the earliest known signal, cWnt, which functions in the posterior half at the beginning of the 8–16 cell-stage [59], is blocked, almost the entire region develops into neurogenic AP [31], suggesting that the initial or pre-signaling fate of sea urchin cells is anterior neuroectoderm. Within this expanded pre-signaling AP, embryos differentiate a number of serotonergic neurons that are scattered throughout the AP, in which other cells are produced with long, immotile, apical tuft cilia [12,13]. Removing Notch signaling from the AP promotes an increased number and the clustering of serotonergic neurons, indicating that lateral inhibition in the AP is another signal that inhibits the serotonergic neural fate in the sea urchin embryo [8]. FoxQ2 is required to exert the serotonergic neural fate initially [12]. However, FoxQ2 is a bifunctional transcription factor that is required early for the specification of most of the cell types in the anterior neuroectoderm, and then it must be removed from the cell late, which subsequently takes on a serotonergic neural fate [8,12–14]. As an initial specifier, the function of FoxQ2 might be similar to that of Zfp521 in mouse embryos. Zfp521 is zygotically expressed by a cell-intrinsic mechanism to exert the initial neural fate [60]. In sea urchins, however, the initial specifier is substituted with a second one, Hbn, because the function of FoxQ2 becomes another one that is against serotonergic neural differentiation after the blastula stage. Taken together, the unknown mechanisms that initially induce *foxQ2* and/or *hbn* at the anterior end of the sea urchin embryo are the substances that determine the initial cell fate, which will be clarified in the future through the analysis of cis-elements of the foxQ2 and hbn genes. So far, none of functional data of Hbn in other systems have been published, but understanding these mechanisms will lead us to answer the question of what is truly the default cell fate in the sea urchin embryo as well as in other organisms.

## Materials and Methods

### Animals and embryo culture

Embryos of *Hemicentrotus pulcherrimus* were collected around Shimoda Marine Research Center, University of Tsukuba, and around the Marine and Coastal Research Center, Ochanomizu University. The divergence time between *H*. *pulcherrimus* used in this study and *S*. *purpuratus* used in most previously described studies was estimated to be 7.2–14 million years ago [61], and the developmental time-course, gene expression patterns, and reported phenotypes in gene-knockdown and/or misexpressed experiments are almost the same. The gametes were collected by the intrablastocoelar injection of 0.5 M KCl, and embryos were cultured in glass beakers or plastic dishes that contained filtered natural seawater (FSW) at 15°C. Cell-permeable JNK inhibitor I, (L)-Form (Merck Millipore, Billerica, MA, USA), was used at 50 μM from the two-cell stage to desired stages [31]. For the control experiment, we added same volume of dimethyl sulfoxide (DMSO), which is used for dissolving the JNK inhibitor.

### Whole-mount *in situ* hybridization and immunohistochemistry

In whole-mount *in situ* hybridization, embryos were fixed with 3.7% formaldehyde-sea water (SW) overnight at 4°C. After 7 x 7 min washes in MOPS buffer (0.1 M MOPS, pH 7.0, 0.5 M NaCl, 0.1% Tween-20), MOPS buffer was substituted with hybridization buffer (HB: 70% formamide, 0.1 M MOPS, pH 7.0, 0.5 M NaCl, 0.1% Tween-20, 1% BSA), and specimens were pre-hybridized at 50°C for 1 h. Subsequently, pre-hybridization HB was substituted with fresh HB containing Dig-labeled RNA probes (0.4 ng/μl final concentration), and samples were incubated at 50°C for 5–7 days. After washing in MOPS buffer for 7 min x 7 times at room temperature (RT), for 1 h x 3 times at 50°C, and for 7 min x 2 times at RT, samples were blocked with 1–5% skim milk (Nacalai Tesque, Tokyo, Japan) in MOPS buffer for 1 h at RT and thereafter incubated with anti-Dig antibody conjugated with alkaline phosphatase (Roche, Basel, Switzerland; 1:1,500 dilution) overnight at 4°C. Tissue was washed with MOPS buffer for a half day with several buffer exchanges. Dig signal was detected with NBT/BCIP (Promega, Madison, WI, USA). For two-color fluorescent *in situ* hybridization, Dig-labeled and FITC-labeled probes were simultaneously applied to HB and detected with anti-Dig and anti-FITC POD-conjugated antibodies, respectively (Roche), followed by the Tyramide signal amplification plus system (TSA-plus; Perkin Elmer, Waltham, MA, USA). After blocking in 1–5% skim milk, specimens were incubated with 1:1,000 diluted anti-Dig POD-conjugated antibody for 1 h at RT, washed with MOPS buffer for 7 min x 7 times at RT, and treated with tetramethylrhodamine TSA-plus for 10 min at RT. Then, samples were washed three times with MOPS buffer, and the remaining POD function was quenched by 0.5% sodium azide in MOPS buffer for 30 min at RT. After washing, we repeated treatment of the samples with anti-FITC antibody and the FITC TSA-plus system. The size of the *foxQ2*-expressing region is quantified with the angle from the posterior end (Fig 3P). The angle was measured using ImageJ and Student’s *t*-test was applied to each quantification to judge whether their differences were significantly meaningful. The graph was drawn with software R [62].

In whole-mount immunohistochemistry, embryos were fixed with 3.7% formaldehyde-SW for 10 min at RT. After washing with PBST (137 mM NaCl, 2.7 mM KCl, 10 mM Na_2_HPO_4_, 1.76 mM KH_2_PO_4_, pH 7.4, 0.1% Tween-20) for 7 min x 7 times, samples were blocked with 1–5% lamb serum in PBST for 1 h at RT and incubated with primary antibodies (dilutions: serotonin (Sigma-Aldrich, St. Louis, MO, USA) 1:2,000, synB [7] 1:100, LRP6 (Sigma) 1:1,000, FoxQ2 [14] 1:100 and c-myc (Sigma) 1:1,000) overnight at 4°C. Antibodies were washed off with PBST for 7 min x 7 times, and the samples were incubated with the secondary antibodies (1:2,000 diluted anti-rabbit IgG conjugated with Alexa 488 and/or 1:2,000 diluted anti-mouse IgG conjugated with Alexa 568 (Thermo Fisher Scientific, Waltham, MA, USA)) for 2 h at RT. The specimens were observed using a Zeiss Axio Imager.Z1 that was equipped with an Apotome system (Zeiss, Oberkochen, Germany) and an Olympus FV10i confocal laser scanning microscope (Olympus, Tokyo, Japan). The optical sections were stacked and analyzed using ImageJ and Adobe Photoshop. Panels and drawings for the figures were made using Microsoft PowerPoint. The number of FoxQ2-positive cells was counted under the fluorescent microscope (IX70, Olympus). Student *t*-test was applied on each quantification to judge whether their differences were significantly meaningful.

### Microinjection of morpholino antisense oligonucleotides (MO) and mRNAs

The morpholino (Gene Tools, Philomath, OR, USA) sequences and the in-needle concentration with 24% glycerol were as follows:

Hbn-MO1 (0.7 mM): 5’- AAAATGAACGGAACAAGTCCAGTGT -3’,

Hbn-MO2 (2.0 mM): 5’- TAGGAGAACCAACGACCGCCGTCAT -3’,

Nodal-MO (0.2 mM): 5’- AGATCCGATGAACGATGCATGGTTA -3’,

Lefty-MO (0.4 mM): 5’- AGCACCGAGTGATAATTCCATATTG -3’,

FoxQ2-MO (0.2 mM): 5’- TCATGATGAAATGTTGGAACGAGAG -3’,

BMP2/4-MO (0.4 mM): 5’- GACCCCAATGTGAGGTGGTAACCAT -3’,

LRP6-MO1 (1.9 mM): 5’- GAAAGGTTTCAAGGCAGCCCATTTC -3’,

LRP6-MO2 (1.5 mM): 5’- TGCCGTTGACTAAATATCATCTACA -3’,

Wnt6-MO1 (3.8 mM): 5’- ACGTGTCCACTCCATCTTGTAATAC -3’,

Wnt6-MO2 (1.9 mM): 5’- TCGTCCAGCGATTTAATAAAGAGCT -3’,

Wnt7-MO1 (3.8 mM): 5’- ATAACCACACCAAgTTgggCCgCAT -3’, and

Wnt7-MO2 (1.9 mM): 5’- GCTCAGCGATGCCCGATGGATAAAA -3’.

Two non-overlapping morpholinos that blocked the translation of Hbn, LRP6, Wnt6 and Wnt7 were used to confirm the specificity of their function. For negative control experiments, we injected 24% glycerol into eggs.

mRNAs were synthesized from linearized plasmids using the mMessage mMachine kit (Thermo Fisher Scientific) and injected at the indicated concentrations in 24% glycerol in needles: hbn-mRNA (0.1 μg/μl), Δ-cadherin (0.3–0.6 μg/μl; [22]), and myc-mRNA (0.1 μg/μl). Microinjections into fertilized eggs and into one blastomere at the two-cell stage were performed as previously described [13].

### Quantitative PCR

Quantitative PCR (qPCR) was performed as previously described [13,63] with some modifications. The total RNA from 100 embryos of *H*. *pulcherrimus* was isolated, and reverse transcription was performed using the Realtime Ready Cell Lysis kit and Transcriptor Universal cDNA Master (Roche). GoTaq qPCR Master Mix (Promega) was used for PCR carried out with a Thermal Cycler Dice Real Time system (Takara, Shiga, Japan). Primer pairs used for qPCR were the following:

Wnt3-qF1; 5’- TATATCCGGCAAACAGGTCC -3’,

Wnt3-qR1; 5’- TCTTCTCCCTCGGAACTGAA -3’,

Wnt6-qF1; 5’- GACCTGCTGGAAGAAAATGC -3’,

Wnt6-qR1; 5’- GGGCTGTTTGACCGTATCAT -3’,

Wnt7-qF1; 5’- CATGGTGTTTCAGGTTCGTG -3’,

Wnt7-qR1; 5’- TCCTAGTTCGTTTGGCCTTG -3’,

COI-qF1; 5’- CCGCATTCTTGCTCCTTCTT -3’, and

COI-qR1; 5’- TGCTGGGTCGAAGAAAGTTG -3’.

The relative concentrations of each mRNA were normalized with mitochondrial COI *C*_*t*_ values.

### Luciferase assay

Top-Flash plasmid M50 Super 8xTOPFlash (Addgene plasmid # 12456) and M51 Super 8xFOPFlash (TOPFlash mutant) (Addgene plasmid # 12457) were gifts from Dr. Randall Moon. DNA fragments containing TCF/LEF-binding sites with Firefly Luciferase gene were amplified by KOD-Fx DNA polymerase (TOYOBO, Tokyo, Japan) with RVprimer3 and EBV_rev_primer set and injected at 20 ng/μl in a needle into the fertilized eggs with a carrier EcoRV-digested *H*. *pulcherrimus* genomic DNA at 10 ng/μl. The signal was obtained from 20–40 embryos for each experiment (three independent batches) using the Bright-Glo Luciferase Assay System (Promega). The luminescence was detected with the LB941 Multimode Reader TriStar (Berthold Technologies GmbH & Co.KG, Bad Wildbad, Germany) for 60 sec. The Top-Flash signal was normalized to the Fop-Flash level for each experiment.

## Supporting Information

S1 Fig Homeobrain is required for the development of serotonergic neurons. (A) Brightfield image of control embryo at 96 h. (B) Serotonin (green) and synB (magenta) in (A). (C) Transmission image in Hbn morphants. (D) Serotonin and synB in Hbn morphants. (E, F) Serotonin and synB in control and HbnMO-2 embryos, respectively, at 72 h. (G) Number of serotonergic neurons in control embryo and Hbn morphants at 72 h. Independent of batches, the number of serotonergic neurons is significantly decreased in Hbn morphants. ***P<0.001, Student’s *t-test*.
(TIF)

S2 Fig Homeobrain mRNA injection partially rescues the deficient effects in its morpholino. (A, B) Similar pattern of neurons in Δcad embryos with or without Hbn mRNA. (C) Without Homeobrain, the serotonergic neurons are not differentiated in Δcad embryos. (D) Exogenous Hbn can partially rescue the Hbn morphant phenotype. (E) Schematic image of the experimental procedure used in 2-cell injections to confirm the sufficiency of Hbn mRNA in the differentiation of serotonergic neurons. (F, G) Control of 2-cell injection experiment. No serotonergic neurons are differentiated in the myc-mRNA alone-injected side of Δcad-Hbn morphants. (H, I) The rescued serotonergic neurons are present at the exogenous Hbn-injected side. (J) The number of serotonergic neurons counted in the half of the embryos in each experiment. The number of serotonergic neurons in the Hbn-mRNA injected side is significantly increased over that in the Hbn-mRNA negative side, but the number does not reach that of the control. *P<0.05, ***P<0.001, Student’s *t-test*.
(TIF)

S3 Fig BMP2/4 is required for the expression of *hbn*. (A-C) *in situ* hybridization reveals that misexpressed Nodal suppresses *hbn*, and qPCR data shows the tendency, but the difference is not significant (<0.5, >2.0) (D). *hbn* mRNA in BMP2/4 morphants is only significantly decreased, indicating that BMP2/4 is required for *hbn* expression.
(TIF)

S4 Fig Spatiotemporal expression pattern of *LRP6* and LRP6 morphants have normal dorsoventral axis. Almost all cells express *LRP6* message in *Hemicentrotus pulcherrimus* from unfertilized egg until at least 25 h (A-C). (D-F) Negative control using sense RNA probe for *LRP6*. (G-J) LRP signal in normal (G-I) and LRP morphants (J). (G) Epifluorescent image of (H). (I) and (J) are stacked images of confocal microscopy and captured under the same microscopic conditions with the same exposure time. (K-O) LRP6 morphants have a normal dorsoventral body axis. Ventral marker, *nodal* (M), and ciliary band marker, *hnf6* (N, O), are normally expressed in LRP6 morphants as they are in the control (K, L). (P) *hbn* disappearance occurs normally in Δcad-LRP6 morphants at 50 h.
(TIF)

S5 Fig Wnt7 is a candidate factor that restricts *foxQ2* expression further to the anterior end when Nodal function is blocked. (A) When Wnt7 is missing, the significant decrease of the *foxQ2* region in Nodal morphants is never occurred, indicating that Wnt7 is the factor that suppresses *foxQ2* in the AP region.*foxQ2* expression patterns in the control (B), Nodal morphants (C), Wnt7 morphant (D) and a Nodal-Wnt7 morphant (E). ***P<0.001, Student’s *t*-test.
(TIF)

S6 Fig Wnt6 but not Wnt7 is required for *hbn* clearance from the anterior end of neuroectoderm. (A-D) The expression pattern of *wnt6* in the control (A, C) and Δcad-embryos (B, D). *wnt6* is slightly expressed at the thickened ectodermal region in Δcad-injected embryos. (E-H) The expression pattern of *wnt7* in the control (E, G) and Δcad-embryos (F, H). (I-K) The expression pattern of *hbn* in Δcad (I), Δcad-Wnt6MO (J) and Δcad-Wnt7MO embryos (K). At this stage, *hbn* is expressed only at the posterior squamous ectoderm and has disappeared from thickened region in Δcad (I) and Δcad-Wnt7MO embryos (K). In contrast, the disappearance of *hbn* is inhibited in Δcad-Wnt6MO embryos (J). Asterisk (*) in (I) indicates the position of squamous ectoderm expressing *hbn*. (L-O) Wnt7 and Wnt6 morphant phenotypes described in the text are reproducible using the second non-overlapped morpholinos for each. *foxQ2* is remains in Wnt7-MO2 morphants at 65 h (M) but not in the control (L). The disappearance of the *hbn* gene from the anterior end of the AP is robust in the control at 65 h (N), but the gene expression is not cleared from the place in Wnt6-MO2 morphants (O). White and black arrowheads indicate the location of *hbn* absence and presence, respectively.
(TIF)

S7 Fig *hbn* clearance at the anterior end is independent of *foxQ2* function. *hbn* expression pattern in the control (A, B, C) and FoxQ2 morphants (D, E, F). *foxQ2* expression pattern in the control (G, H) and Hbn morphants (I, J). These results indicate that their expression is mutually independent. MBL, mesenchyme blastula; early G, early gastrula.
(TIF)

S8 Fig The exogenous mRNA is degraded by 24 h in sea urchin embryos. mRNA encoding Hbn was injected into fertilized eggs and detected with *in situ* hybridization using the Hbn probe. The exogenous mRNA was detected at 16 h in the whole body (A), but by 24 h only the endogenous *hbn* was detected (B).
(TIF)
