# Supplementary figures and images for: Cooperative Wnt-Nodal Signals Regulate the Patterning of Anterior Neuroectoderm
Source: PLoS Genet. 2016 Apr 21;12(4):e1006001. doi: 10.1371/journal.pgen.1006001 (PMC4839626; doi:10.1371/journal.pgen.1006001)

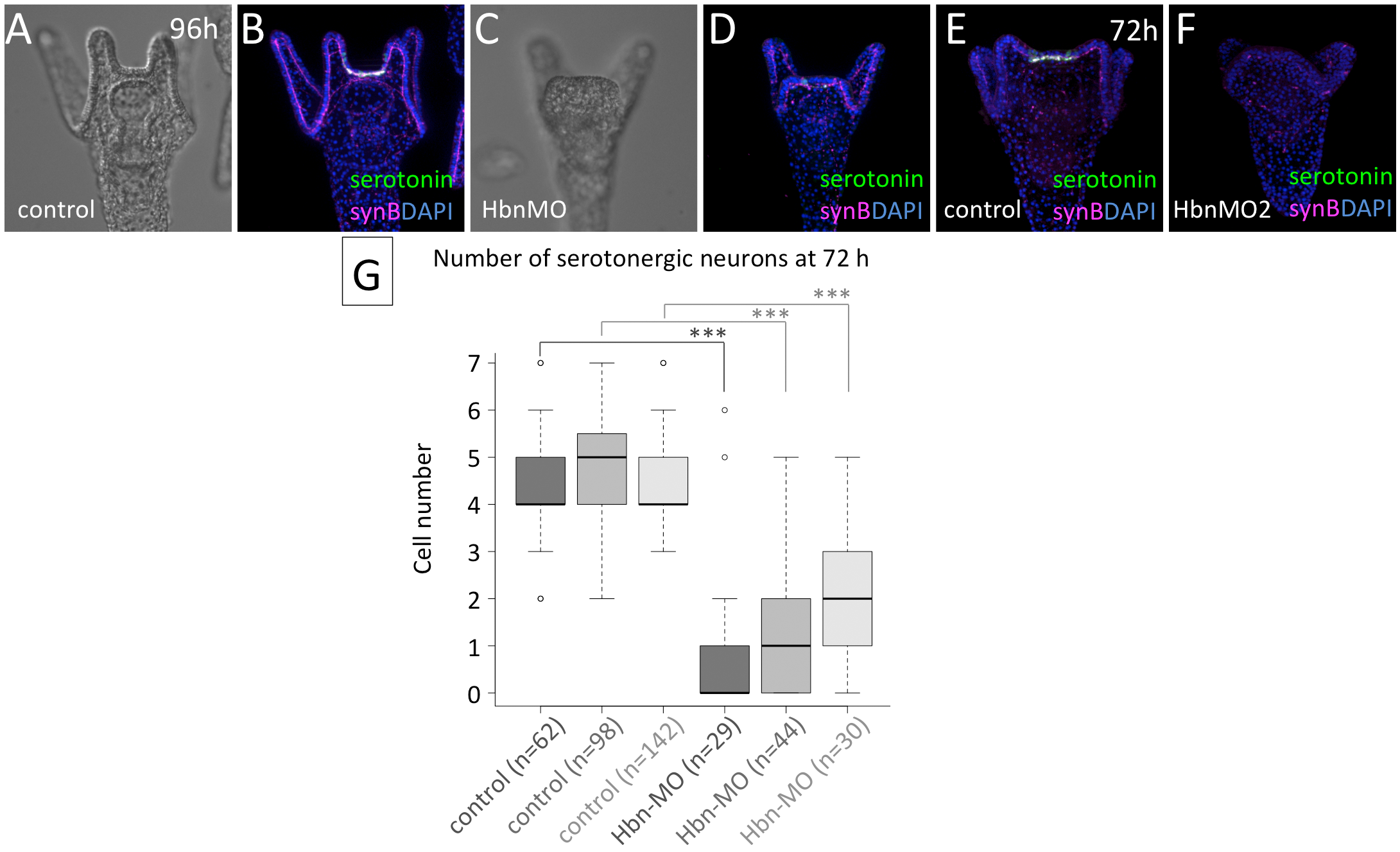

Supplement: S1 Fig — (A) Brightfield image of control embryo at 96 h. (B) Serotonin (green) and synB (magenta) in (A). (C) Transmission image in Hbn morphants. (D) Serotonin and synB in Hbn morphants. (E, F) Serotonin and synB in control and HbnMO-2 embryos, respectively, at 72 h. (G) Number of serotonergic neurons in control embryo and Hbn morphants at 72 h. Independent of batches, the number of serotonergic neurons is significantly decreased in Hbn morphants. ***P<0.001, Student’s t-test. (TIF) [file pgen.1006001.s001.tif]

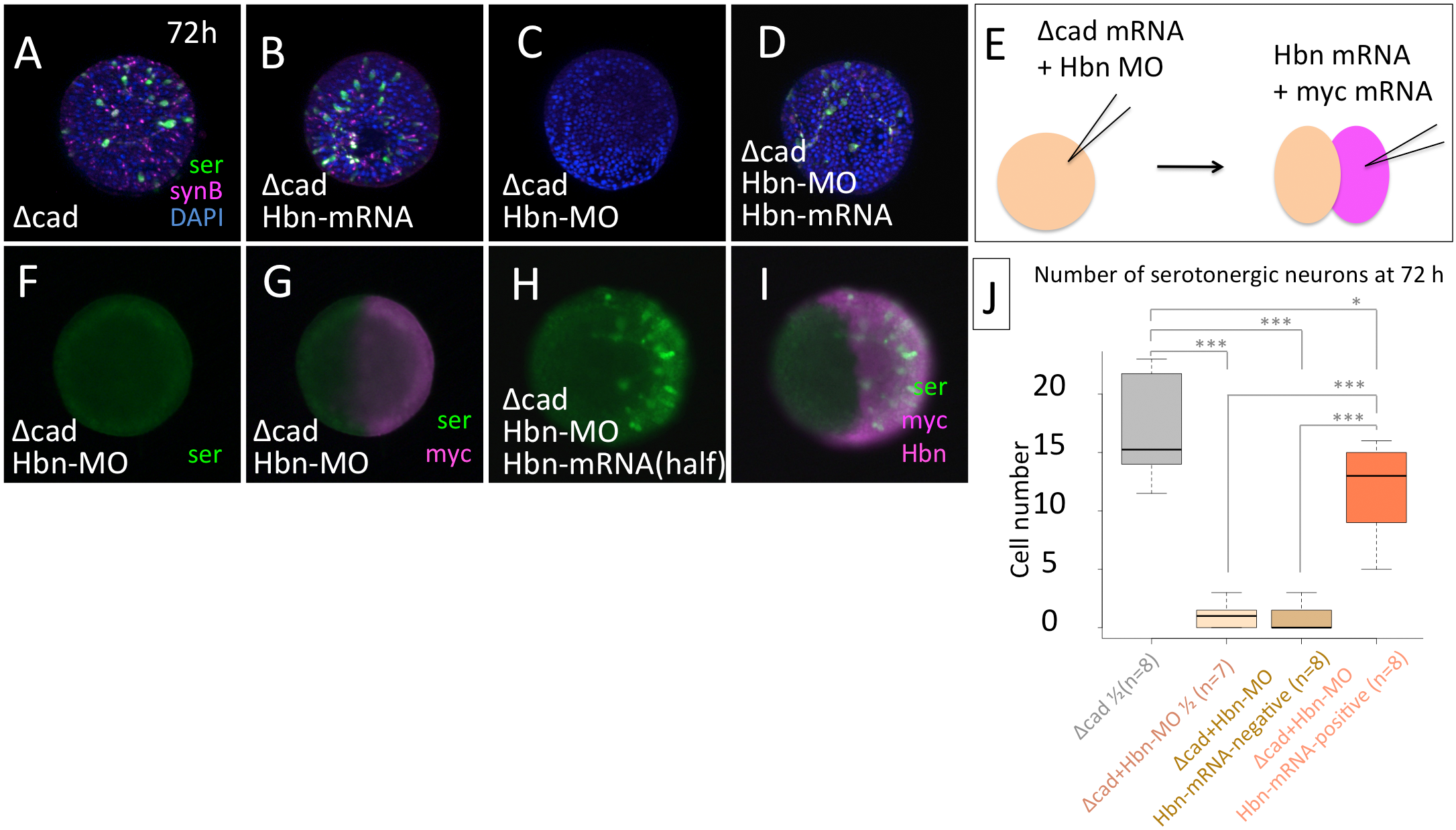

Supplement: S2 Fig — (A, B) Similar pattern of neurons in Δcad embryos with or without Hbn mRNA. (C) Without Homeobrain, the serotonergic neurons are not differentiated in Δcad embryos. (D) Exogenous Hbn can partially rescue the Hbn morphant phenotype. (E) Schematic image of the experimental procedure used in 2-cell injections to confirm the sufficiency of Hbn mRNA in the differentiation of serotonergic neurons. (F, G) Control of 2-cell injection experiment. No serotonergic neurons are differentiated in the myc-mRNA alone-injected side of Δcad-Hbn morphants. (H, I) The rescued serotonergic neurons are present at the exogenous Hbn-injected side. (J) The number of serotonergic neurons counted in the half of the embryos in each experiment. The number of serotonergic neurons in the Hbn-mRNA injected side is significantly increased over that in the Hbn-mRNA negative side, but the number does not reach that of the control. *P<0.05, ***P<0.001, Student’s t-test. (TIF) [file pgen.1006001.s002.tif]

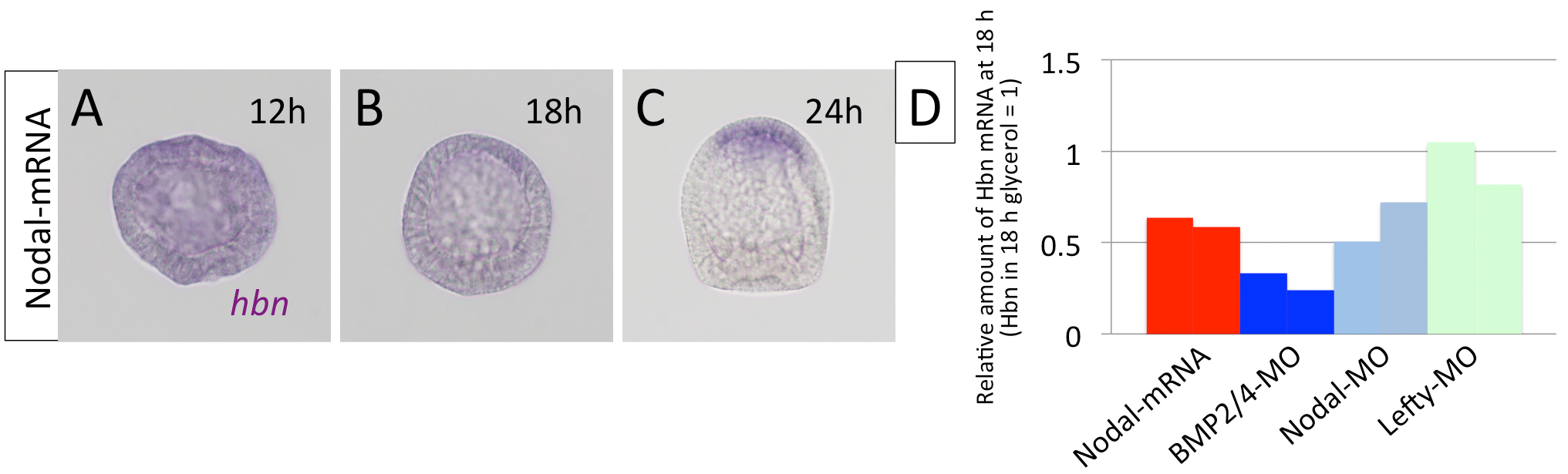

Supplement: S3 Fig — (A-C) in situ hybridization reveals that misexpressed Nodal suppresses hbn, and qPCR data shows the tendency, but the difference is not significant (<0.5, >2.0) (D). hbn mRNA in BMP2/4 morphants is only significantly decreased, indicating that BMP2/4 is required for hbn expression. (TIF) [file pgen.1006001.s003.tif]

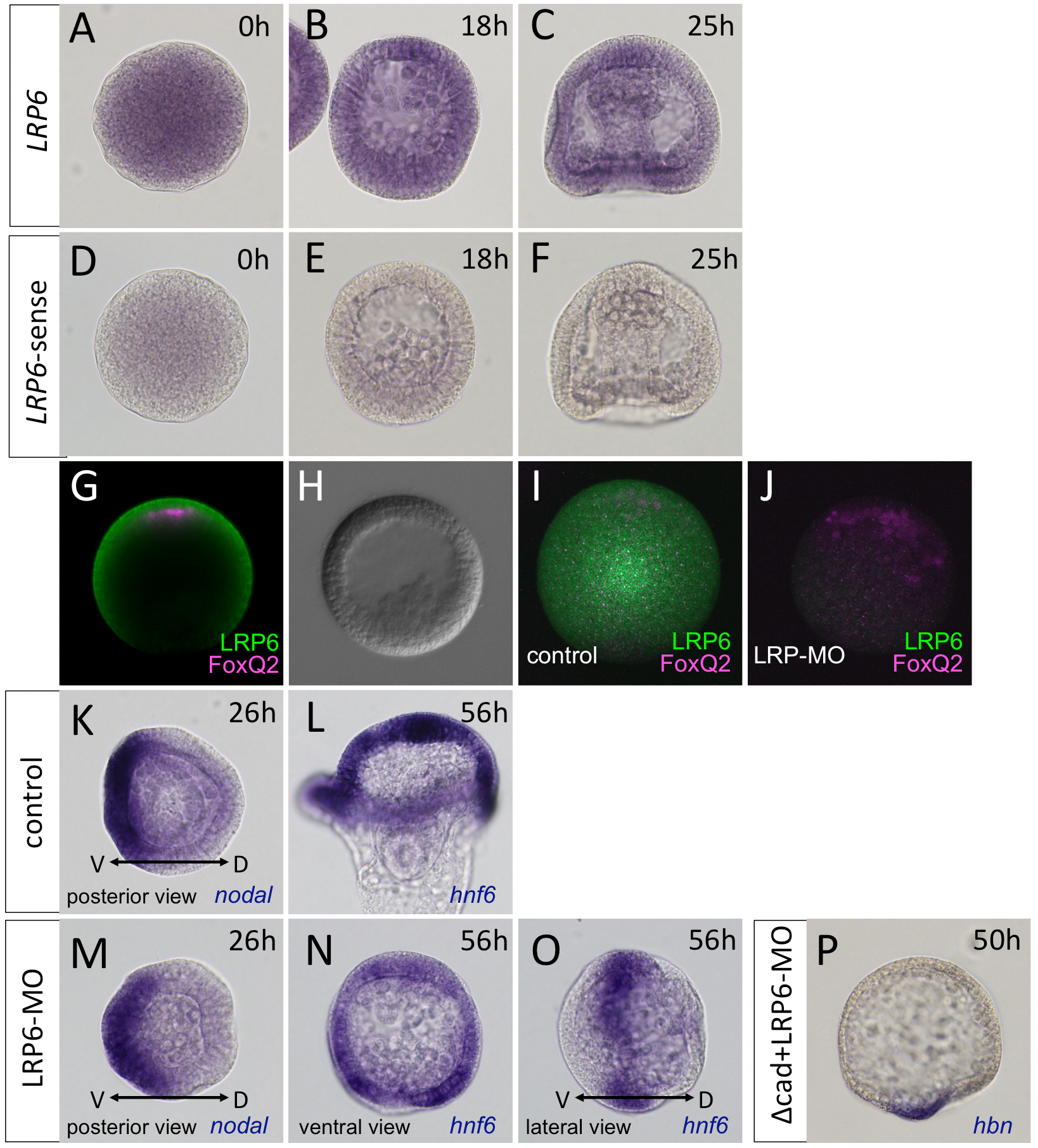

Supplement: S4 Fig — Almost all cells express LRP6 message in Hemicentrotus pulcherrimus from unfertilized egg until at least 25 h (A-C). (D-F) Negative control using sense RNA probe for LRP6. (G-J) LRP signal in normal (G-I) and LRP morphants (J). (G) Epifluorescent image of (H). (I) and (J) are stacked images of confocal microscopy and captured under the same microscopic conditions with the same exposure time. (K-O) LRP6 morphants have a normal dorsoventral body axis. Ventral marker, nodal (M), and ciliary band marker, hnf6 (N, O), are normally expressed in LRP6 morphants as they are in the control (K, L). (P) hbn disappearance occurs normally in Δcad-LRP6 morphants at 50 h. (TIF) [file pgen.1006001.s004.tif]

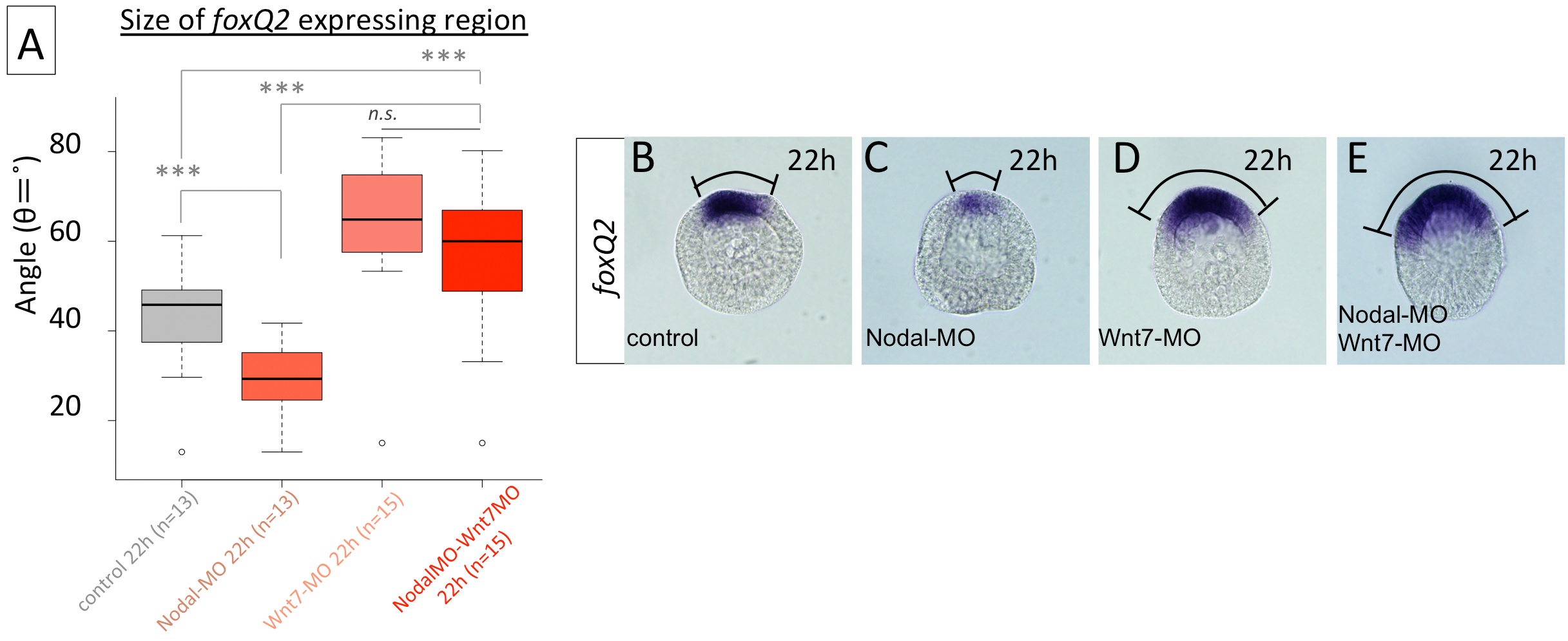

Supplement: S5 Fig — (A) When Wnt7 is missing, the significant decrease of the foxQ2 region in Nodal morphants is never occurred, indicating that Wnt7 is the factor that suppresses foxQ2 in the AP region.foxQ2 expression patterns in the control (B), Nodal morphants (C), Wnt7 morphant (D) and a Nodal-Wnt7 morphant (E). ***P<0.001, Student’s t-test. (TIF) [file pgen.1006001.s005.tif]

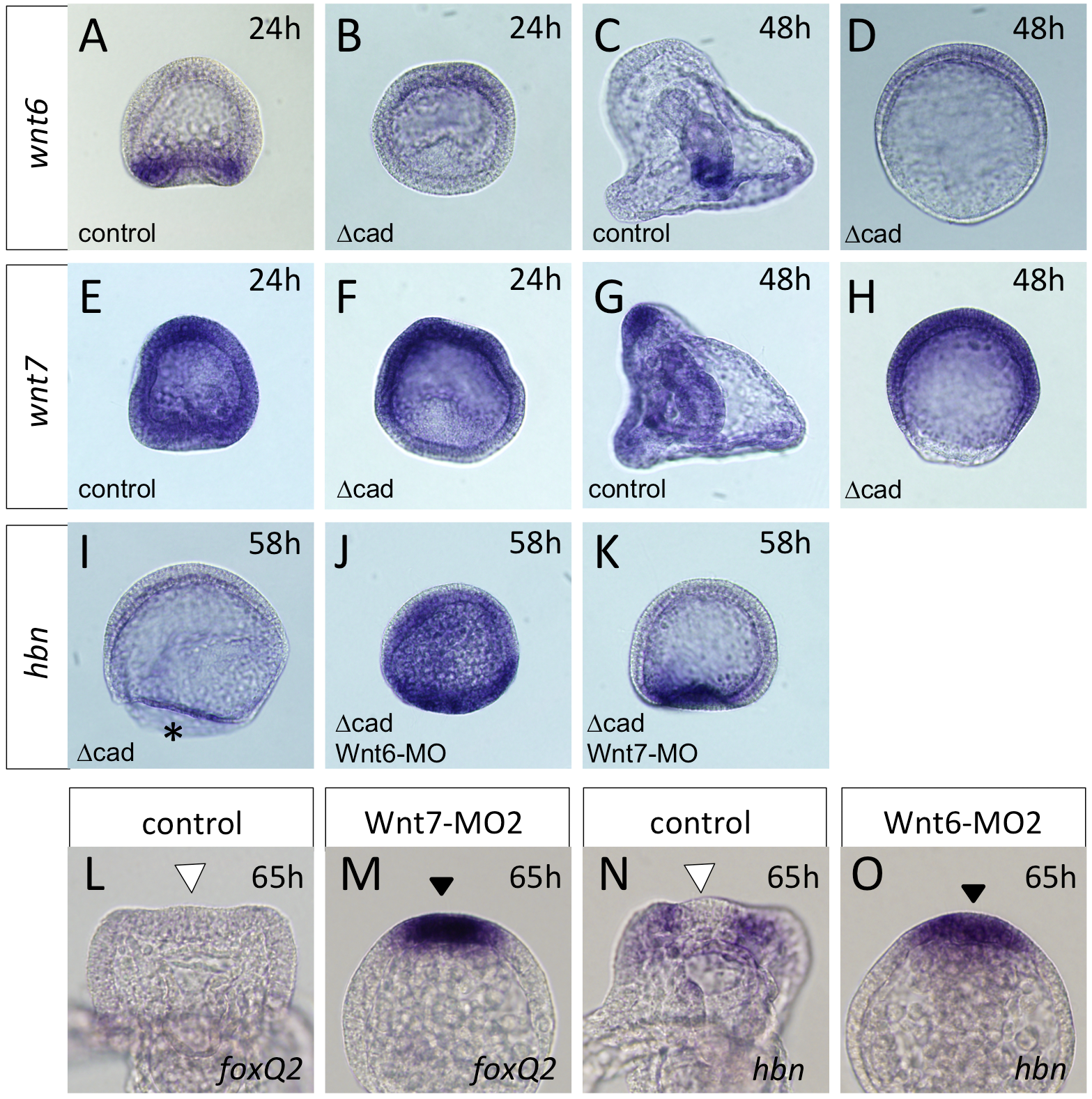

Supplement: S6 Fig — (A-D) The expression pattern of wnt6 in the control (A, C) and Δcad-embryos (B, D). wnt6 is slightly expressed at the thickened ectodermal region in Δcad-injected embryos. (E-H) The expression pattern of wnt7 in the control (E, G) and Δcad-embryos (F, H). (I-K) The expression pattern of hbn in Δcad (I), Δcad-Wnt6MO (J) and Δcad-Wnt7MO embryos (K). At this stage, hbn is expressed only at the posterior squamous ectoderm and has disappeared from thickened region in Δcad (I) and Δcad-Wnt7MO embryos (K). In contrast, the disappearance of hbn is inhibited in Δcad-Wnt6MO embryos (J). Asterisk (*) in (I) indicates the position of squamous ectoderm expressing hbn. (L-O) Wnt7 and Wnt6 morphant phenotypes described in the text are reproducible using the second non-overlapped morpholinos for each. foxQ2 is remains in Wnt7-MO2 morphants at 65 h (M) but not in the control (L). The disappearance of the hbn gene from the anterior end of the AP is robust in the control at 65 h (N), but the gene expression is not cleared from the place in Wnt6-MO2 morphants (O). White and black arrowheads indicate the location of hbn absence and presence, respectively. (TIF) [file pgen.1006001.s006.tif]

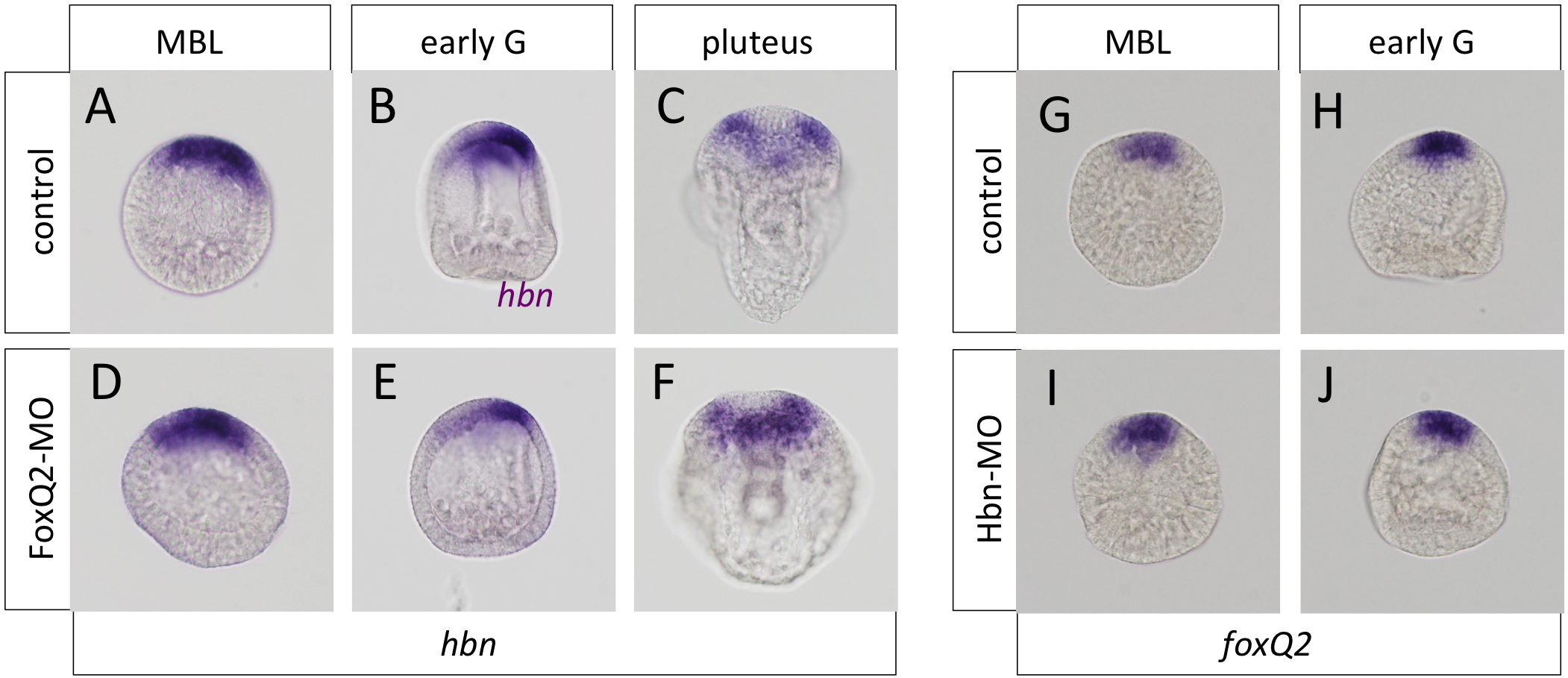

Supplement: S7 Fig — hbn expression pattern in the control (A, B, C) and FoxQ2 morphants (D, E, F). foxQ2 expression pattern in the control (G, H) and Hbn morphants (I, J). These results indicate that their expression is mutually independent. MBL, mesenchyme blastula; early G, early gastrula. (TIF) [file pgen.1006001.s007.tif]

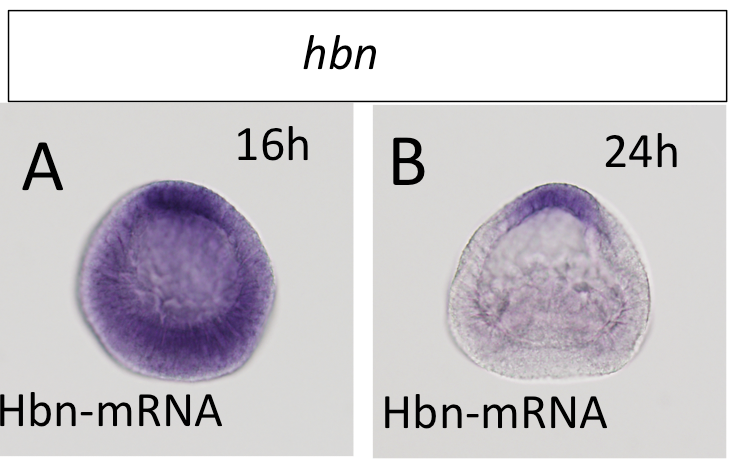

Supplement: S8 Fig — mRNA encoding Hbn was injected into fertilized eggs and detected with in situ hybridization using the Hbn probe. The exogenous mRNA was detected at 16 h in the whole body (A), but by 24 h only the endogenous hbn was detected (B). (TIF) [file pgen.1006001.s008.tif]
